# Single-cell profiling uncovers the intricate pathological niche diversity in brain, lymph node, bone, and adrenal metastases of lung cancer

**DOI:** 10.1007/s12672-025-02269-w

**Published:** 2025-04-10

**Authors:** Le Liu, Yuan Zhou, Zhenjun Ye, Zhiyong Chen, Benchao Yuan, Liyi Guo, Haiyan Zhang, Yuanyuan Xu

**Affiliations:** 1 Huizhou Sixth People’s Hospital, Huizhou, 516211 China; 2 Lanzhou University Second Hospital, Lanzhou, 730000 China

**Keywords:** Lung cancer metastasis, Pathological niches, Immune microenvironment, ScRNA-seq, Functional heterogeneity

## Abstract

**Purpose:**

The aim of this study is to explore the pathological niche of cancer metastasis and the site-specific interactions between tumor cells and the microenvironment, understand the mechanisms driving metastasis progression and identify potential therapeutic targets.

**Methods:**

Data from four lung cancer metastasis datasets (GSE123902, GSE131907, GSE148071, and GSE186344) were downloaded and subjected to stringent quality control and filtering. Cell types were identified using canonical markers, and pseudotime trajectory analysis was performed to evaluate cell differentiation. Functional and pathway enrichment analyses, including ssGSEA and GO/KEGG, were conducted. CellphoneDB was used to analyze intercellular communication, ranking receptor-ligand interactions based on communication strength.

**Results:**

Eleven cell types were identified after quality control, revealing significant heterogeneity and site-specific functionality in lung cancer metastases. CTLs showed notable activity in antigen presentation and T-cell differentiation pathways, with DNAJB1⁺ CTLs playing a dominant role in cytotoxicity and immune regulation. B cells, myeloid cells, and CAFs were involved in immune modulation, defense, and matrix remodeling through specific signaling pathways. Tumor cell subclusters drove proliferation, migration, and immune evasion via immune-regulatory, Hippo, and TGF-beta pathways. No overlapping pathways were observed across metastatic sites. Cell communication analysis identified PPIA-BSG and APP-CD74 as key axes in brain and lymph node metastases, while FN1-Integrin and CTLA4-CD86 dominated in bone and adrenal metastases, respectively.

**Conclusions:**

In summary, this study highlights the functional heterogeneity and site-specific interactions of cells in lung cancer metastases, providing insights into the mechanisms shaping metastatic niches and potential therapeutic strategies.

**Supplementary Information:**

The online version contains supplementary material available at 10.1007/s12672-025-02269-w.

## Introduction

Lung cancer stands as one of the leading causes of cancer-related mortality globally [1]. Despite significant advancements in early detection and therapeutic interventions, the prognosis for patients with metastatic lung cancer remains dismal. A major challenge in treating lung cancer is its ability to metastasize and form diverse pathological niches in different organs, each with its unique microenvironment, which influences tumor progression and treatment resistance [2].

The pathological niches of tumors are the products of intricate interactions between tumor cells and their surrounding microenvironment, where the interplay between immune cells and tumor cells plays a pivotal role [3, 4]. Immune cells not only regulate tumor growth, metastasis, and immune evasion through their interactions but also exert profound influence on the tumor microenvironment's architecture. For instance, tumor-associated macrophages (TAMs) expressing high levels of *CD36* acquire immunosuppressive properties that facilitate tumor progression [5]. Similarly, NNMT⁺ cancer-associated fibroblasts (CAFs) drive tumor cell proliferation and resistance to PD-L1 blockade immunotherapy by recruiting TAMs through epigenetic reprogramming involving serum amyloid A [6]. On the other hand, pancreatic cancer cells with high surface expression of specific proteins enhance the formation of robust immunological synapses between cytotoxic T lymphocytes (CTLs) and CLDN18⁺ cancer cells, thereby boosting T cell activation [7]. Conversely, tumor cells can also modulate other immune cells to evade immune surveillance. For example, malignant skin CTCL cells exploit the immune gene programs of T helper 2 cells and mimic the tumor microenvironment to sustain their survival [8]. Despite extensive research uncovering the roles of immune cells in the tumor microenvironment, studies focusing on the distinct immunological features of metastatic niches remain relatively limited. The pronounced microenvironmental heterogeneity among metastatic sites further underscores this gap [9]. Brain, lymph node, bone, and adrenal gland metastases represent the most common and clinically challenging sites of lung cancer dissemination, characterized by high invasiveness, immune evasion, and therapeutic resistance. For instance, a comprehensive analysis by Duan et al. revealed that primary lung tumors and brain metastases consistently share a set of gene mutations [10]. Hence, investigating the unique immune response mechanisms and microenvironmental characteristics of these metastatic niches is essential for deepening our understanding of lung cancer metastasis and developing innovative therapeutic strategies.

This study seeks to systematically dissect the cellular composition, functional specialization, and intercellular dynamics within lung cancer metastases through single-cell RNA sequencing (scRNA-seq). Leveraging four metastasis datasets, we utilized pseudotime trajectory analysis, pathway enrichment, and cell communication profiling to unravel the intricate interactions between immune and tumor cells and the metabolic adaptations that define the immune microenvironment of metastatic niches.

## Methods

### Data collection and pre-processing

We retrieved four single-cell datasets from the GEO database, corresponding to lung cancer metastases in the brain, lymph nodes, bones, and adrenal glands, specifically GSE123902, GSE131907, GSE148071, and GSE186344 [11–14].The raw data underwent quality control, removing cells with fewer than 100 genes, more than 6000 genes, or a mitochondrial gene expression ratio exceeding 20%. The filtered data were subsequently normalized, and principal component analysis (PCA) was performed using the 2000 most variable genes. Batch effects were corrected using the Harmony algorithm. Dimensionality reduction was achieved through UMAP, followed by clustering of cells with the Leiden algorithm. Finally, cell types were annotated based on marker genes. All analyses were conducted in Python (v3.8.12) using the following libraries: pandas (v1.5.3), numpy (v1.23.5), scanpy (v1.9.6), anndata (v0.9.2), and matplotlib (v3.5.2).

### Differential expression analysis

“rank_gene_groups” was applied using the Student’s t-test with default parameters, and p-values were adjusted using the Benjamini–Hochberg method. DEGs were defined as genes with an adjusted p-value below 0.05 and an average log2 fold change greater than 1. Cell types for each cluster were annotated using canonical markers identified within the DEGs, based on data from the CellMarker2 database. Marker expression for each cell type was visualized using the clustermap and dotplot functions from Seaborn v3.1.2.

### Pathway enrichment analysis

GO and KEGG analyses were performed to investigate the potential biological functions and associated signaling pathways for each cell type. The Metascape online platform was employed to carry out these analyses, with only biological process categories used as the background set for GO analysis. Pathways or functions with adjusted p-values below 0.05 were deemed significantly enriched. Additionally, single-sample GSEA was conducted using all pathway sets from MSigDB through gseapy (v1.1.3), with gene expression levels in individual cells provided as input data.

### ssGSEA analysis

We applied ssGSEA to assess gene set activity for each sample. Relevant gene sets, such as Hallmark and KEGG pathways, were selected from public databases. A normalized gene expression matrix was extracted from single-cell data to ensure comparability between samples. SSGSEA analysis was performed using R (version 4.0 or higher) and the GSVA package (version 1.34.0), with the ssGSEA function used to calculate enrichment scores (ES) for each sample, representing the activity level of the gene sets. The results were visualized through heatmaps.

### Pseudotime inference

Pseudotime trajectory analysis was performed using the Palantir algorithm to track the differentiation and transitions among cell subtypes. From each cluster, the top eight genes with the highest variability were identified using the Scanpy v1.9.6 function “rank_gene_groups” and utilized for trajectory construction. UMAP was applied for dimensionality reduction, and the plot_trajectory tool was employed to visualize the resulting trajectories.

### Cell communication analysis

Cell–cell communication between immune/stromal cells and cancer cells was analyzed using known ligand-receptor pairs through CellphoneDB version 5.0.1. To calculate the null distribution of average ligand-receptor pair expression, 1000 permutations were applied to randomized cell identities. Expression thresholds for individual ligands or receptors were determined across cell types based on the distribution of average log-transformed gene expression.

### Statistical analysis

Statistical analyses were performed using the scipy.stats.ttest_ind function, with results presented as means ± standard deviation (SD). Boxplots indicate the median (center line), the 25 th and 75 th percentiles (box edges), and the minimum and maximum values (whiskers). Differences in immune checkpoint expression were evaluated using one-way ANOVA, with significance levels indicated as follows: ***P < 0.001; **P < 0.01; *P < 0.05.

## Results

### scRNA-seq initially characterizes the pathological niches in different lung cancer metastases

**Fig. 1:**
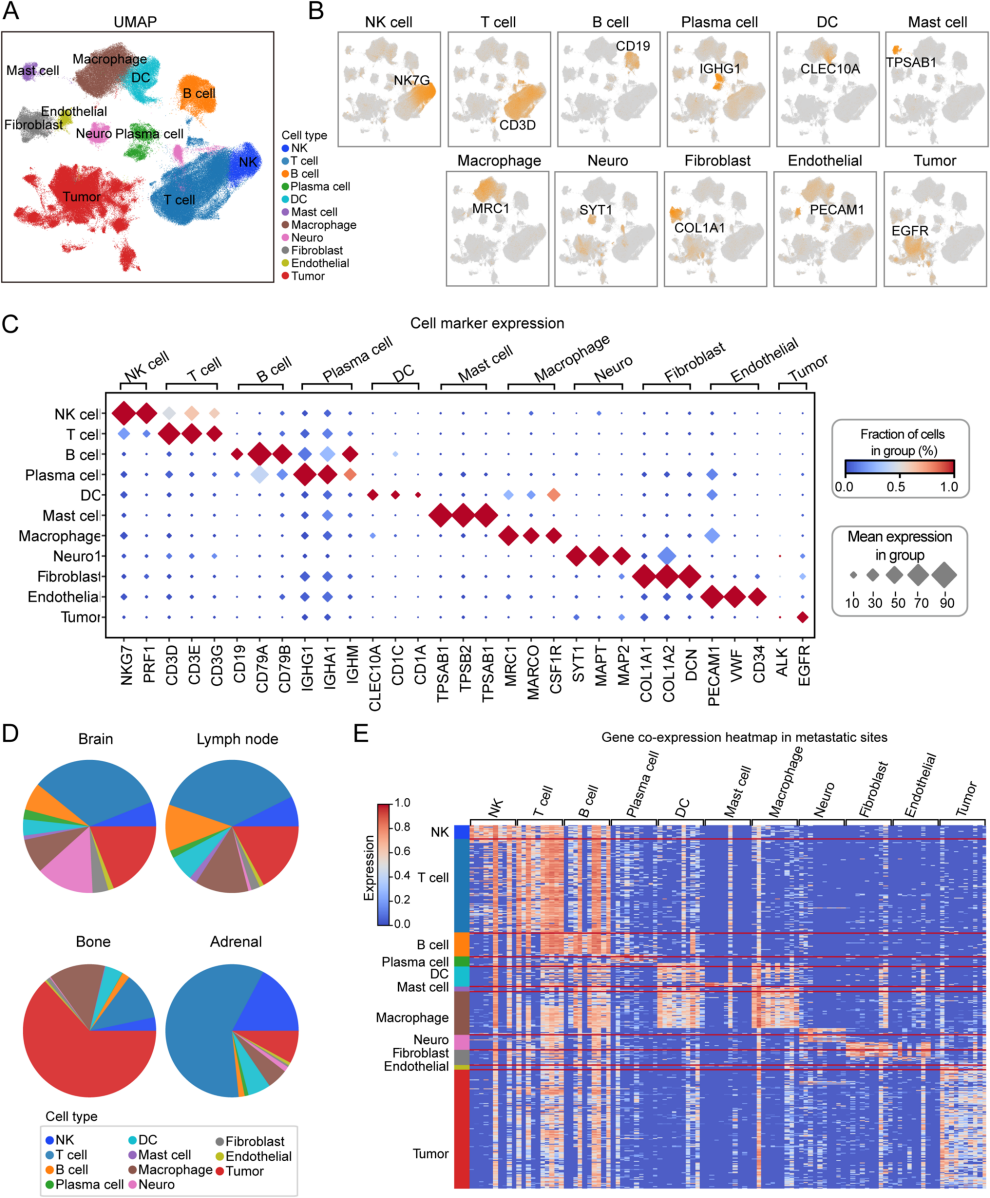
Profiling the immune microenvironment of lung cancer brain, lymph node, bone, and adrenal metastases through single-cell transcriptomics.** A** UMAP plots showing all single cells, with 11 cell types distinctly color-coded. **B** Expression patterns of characteristic marker genes for each cell type: NK cells (*NK7G*), T cells (*CD3D*), B cells (*CD19*), Plasma cells (*IGHG1*), Dendritic cells (*CLEC10 A*), Mast cells (*TPSAB1*), Macrophages (*MRC1*), Neurons (*SYT1*), Fibroblasts (*COL1 A1*), Endothelial cells (*PECAM1*), and Tumor cells (*EGFR*). **C** Dot plot illustrating the expression levels of key marker genes across cell types. **D** Pie charts depicting the proportions of each cell type across brain, lymph node, bone, and adrenal gland metastatic sites. **E** Heatmap of the top ten highly expressed differential genes across all cell types in the four metastatic sites of lung cancer

As the starting point of tumor pathological ecological niche research, we initially performed quality control and filtering on the cells from four datasets collected from metastatic lesions, resulting in 358,124 high-quality cells. Following standardization and batch effect correction, we conducted unsupervised clustering analysis to identify the major cell clusters with similar gene expression patterns (Fig. S1). Through these analyses, we successfully identified 11 distinct cell types, and their identities were confirmed based on classical biological markers and the high expression of genes with the greatest variation in the transcriptome (Fig. 1A). These cell types include NK cells (*NK7G*), T cells (*CD3D*), B cells (*CD19*), plasma cells (*IGHG1*), dendritic cells (*CLEC10 A*), mast cells (*TPSAB1*), macrophages (*MRC1*), neurons (*SYT1*), fibroblasts (*COL1 A1*), endothelial cells (*PECAM1*), and tumor cells (*EGFR*) (Fig. 1B). The marker genes for each cell cluster are depicted in a dot plot (Fig. 1C).

Furthermore, we analyzed the proportions of different cell types across the four metastatic lesions and observed significant differences in cell distributions between them (Fig. 1D). For example, T cells were most abundant in the brain, lymph nodes, and adrenal gland metastases, whereas tumor cells were most prevalent in bone metastasis, followed by T cells and mast cells. These findings suggest that the differential distribution of cell types between metastatic lesions plays a key role in shaping the unique immune microenvironment at each site. Moreover, the distinct gene expression profiles exhibited by different cell types in each metastatic lesion (Fig. 1E) may be closely associated with the formation of the tumor microenvironment and tumor progression.

### Diverse functional roles of T cells in distinct metastatic immune microenvironments

**Fig. 2:**
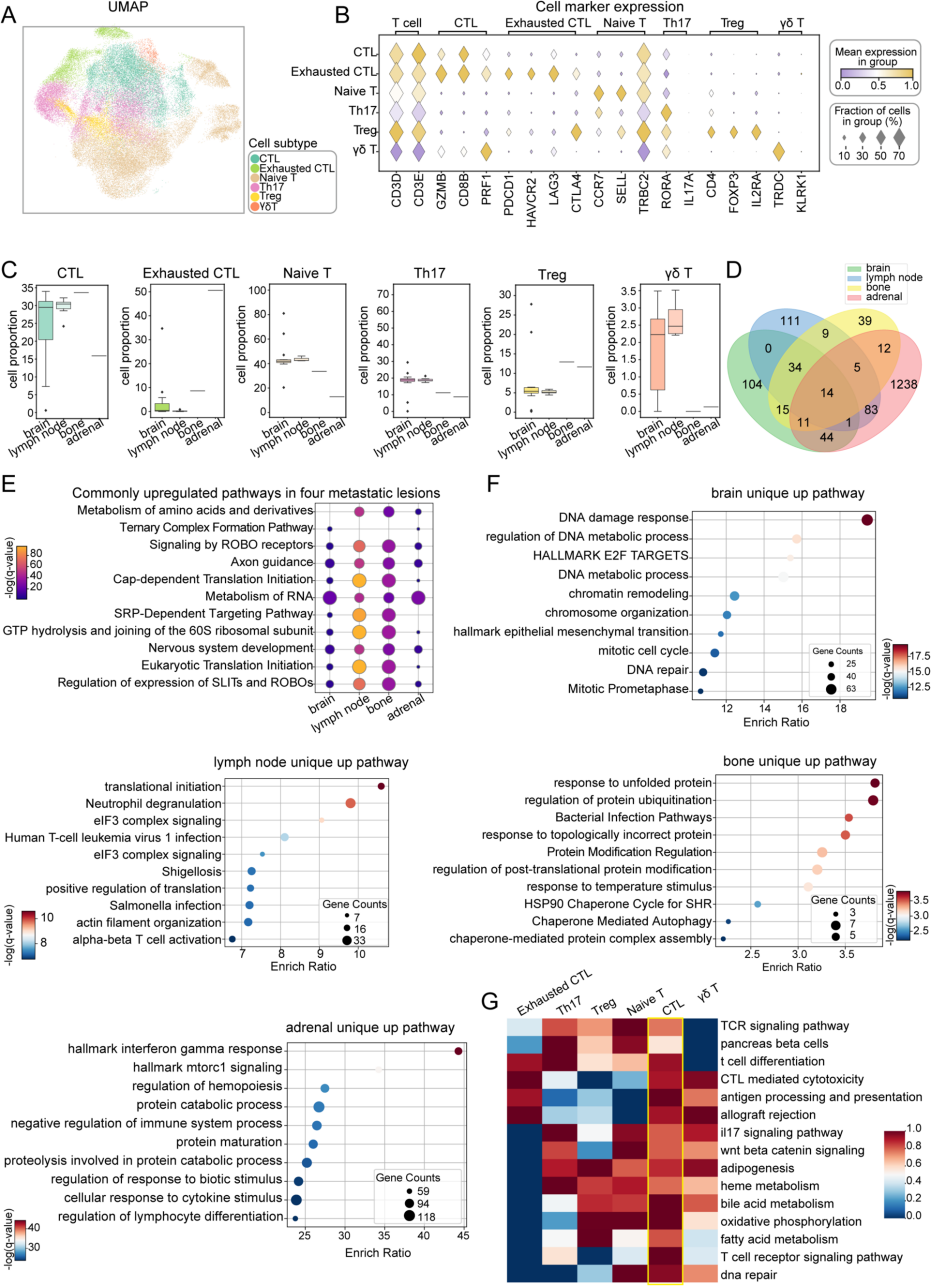
T cells heterogeneity in four metastatic foci.** A** UMAP plot displaying T cell subtypes. **B** Dot plot showing the expression of cell-specific marker genes across T cell subtypes. **C** Boxplots illustrating the proportions of each T cells in metastatic sites, including brain, lymph node, bone, and adrenal gland. **D** Venn diagram of pathways enriched for genes highly expressed in the cells relative to the other three metastatic sites. **E** T cells upregulation of gene co-expression pathways in four metastases. **F** Pathway enrichment specific to T cells in brain, lymph node, bone, and adrenal metastases. **G** ssGSEA pathway scores for T cells, with redder colors indicating higher pathway activity

During the process of tumor metastasis, the dynamic remodeling of the immune microenvironment resembles a complex ecological network, with T cells emerging as pivotal regulators. Our analysis focused on T cells, which play a crucial role in mediating tumor immunity and shaping the immune response in cancer. Through reclustering analysis, we identified six distinct T cell subtypes from metastatic lesions across patients (Fig. 2A). These subtypes exhibit unique transcriptional"signatures,"as illustrated by the UMAP plot in Fig. S2A and the dot plot in Fig. 2B. The proportions of these T cell subtypes vary significantly across metastatic sites (Fig. 2C). Notably, CTLs are consistently enriched in all metastatic lesions, underscoring their critical role in tumor immunity. Meanwhile, exhausted CTLs are most prevalent in adrenal metastases, highlighting potential site-specific functional adaptations. To elucidate the functional heterogeneity of T cells across metastatic sites, we performed pathway enrichment analysis based on site-specific highly expressed genes (Fig. S2B). The results revealed that while T cells across all metastatic sites shared 14 core pathways, each site also exhibited distinct enriched pathways (Fig. 2D, E). For instance, T cells in brain metastases demonstrated pronounced activity in the DNA damage response pathways, whereas those in lymph node metastases were predominantly involved in the translational initiation pathway. In bone metastases, T cells responded to unfolded protein stress and protein ubiquitination regulation, while T cells in adrenal metastases displayed enrichment in the Hallmark Interferon Gamma Response pathway. These findings indicate that T cells assume diverse functional roles within the unique immune microenvironments of metastatic lesions (Fig. 2F).

Although the functional characteristics of T cells vary across metastatic sites, further dissection of T cell subtypes, particularly CTLs, revealed that CTLs exhibit pronounced activity across numerous immune-related pathways. For example, pathways related to antigen presentation, CTL-mediated cytotoxicity, and T cell differentiation all showed high functional scores (Fig. 2G). These findings suggest that CTLs may act as a central subset in immune responses, actively regulating anti-tumor immunity through multiple pathways in metastatic lesions.

### Functional specialization of CTL subtypes highlights their roles in shaping the immune microenvironment of lung cancer metastases

**Fig. 3:**
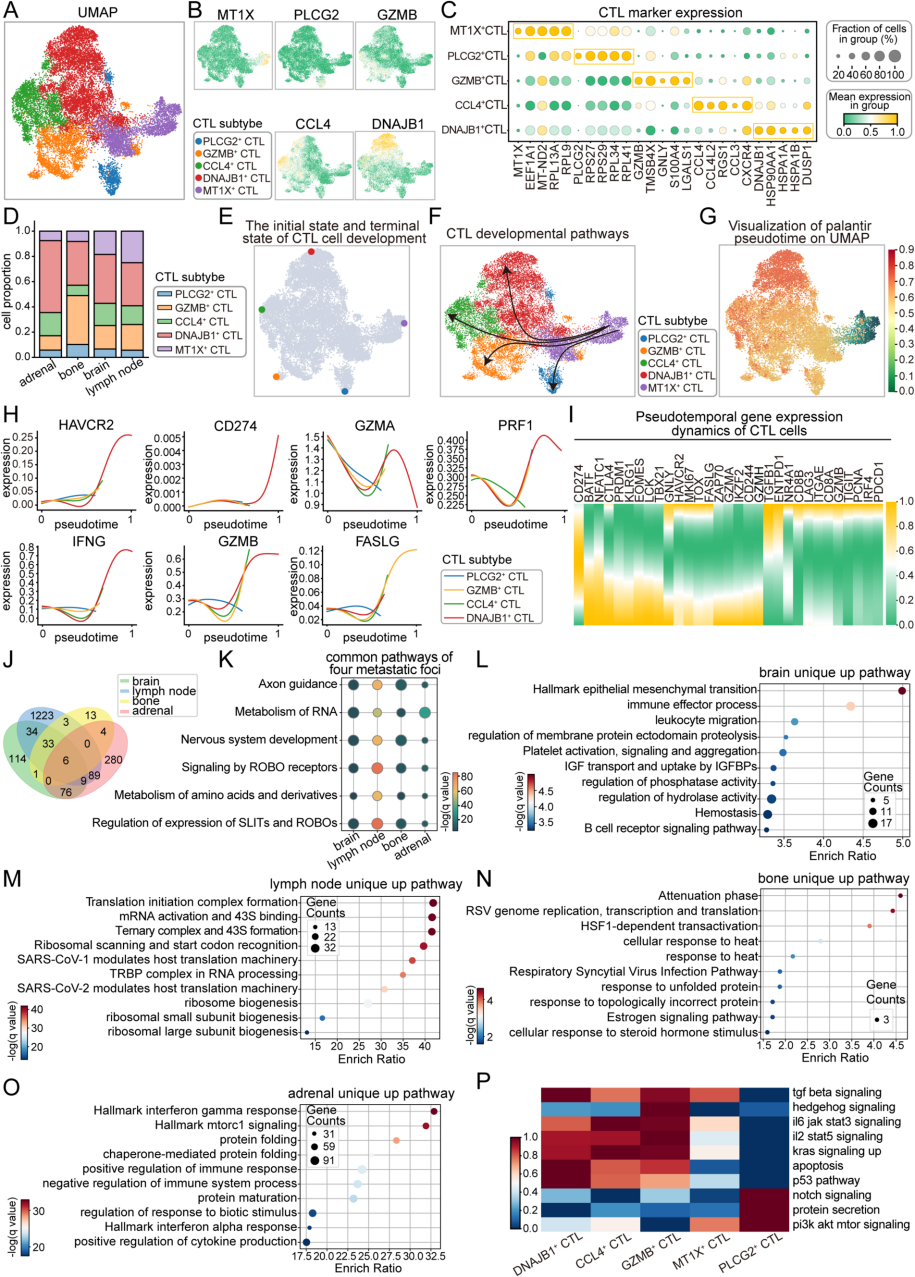
Development and heterogeneity of CTLs in metastatic sites. **A** UMAP plot of CTL subtypes, with different colors representing distinct cell subtypes, and one specific subtype highlighted. **B** Expression of marker genes for CTL subtypes, including *MI1X*, *PLCG2*, *GZMB*, *CCL4*, and *DNAJB1*. **C** Dot plot showing the expression levels of typical genes for CTL subtypes. **D** Bar chart showing the proportions of different CTL subtypes across four metastatic sites. **E** Schematic representation of the initial and terminal states of CTL subtype development, with the MT1X^+^CTL subtype defined as the developmental starting point, and other subtypes as developmental endpoints. **F** Schematic diagram illustrating the developmental trajectory of CTL subtypes from the starting point to the endpoint. **G** Visualization of initial pseudotime on UMAP, with different colors representing the various stages of cell development. **H** Pseudotime expression dynamics of *HAVCR2*, *CD274*, *GZMA*, *FASLG*, *IFNG*, *GZMB* and *PRF1* genes across the four subtypes. **I** Heatmap of pseudotime gene expression dynamics in CTL subtypes, with color intensity representing gene expression levels. **J** Venn diagram of pathways enriched for highly expressed genes in CTLs across the four metastatic sites. **K** Six pathways commonly enriched in CTLs across the four metastatic sites. **L–O** CTL-specific pathways in brain, lymph node, bone, and adrenal metastases. **P** ssGSEA pathway scores for CTL subsets, with redder colors indicating higher pathway activity

Based on the high expression of specific marker genes, we identified five distinct CTL subtypes. These subtypes were characterized by their differential expression of known CTL markers (Figs. 3A–C, S3A). By comparing the proportional distribution of these subtypes across different metastatic sites, we observed that DNAJB1⁺ CTL accounted for a significant proportion in all four metastatic sites (Fig. 3D). Interestingly, in bone metastases, the proportion of DNAJB1⁺ CTL was comparable to that of GZMB⁺ CTL, showcasing distinct immunological distribution patterns.

To further investigate the developmental trajectories, differentiation processes, and responses to external stimuli of CTL subtypes in lung cancer metastases, we defined MT1X⁺ CTLs as the developmental starting point due to their high expression marking the early stages of immune response activation [15, 16], while other subtypes were considered endpoints (Fig. 3E–G). By analyzing developmental pathways and visualizing key gene expression, we observed a progressive upregulation of critical genes in DNAJB1⁺ CTL and GZMB⁺ CTL (Fig. 3H). These included immune checkpoint genes (e.g., *HAVCR2*), cell surface molecules (e.g., *CD274*), cytotoxic effector molecules (e.g., *GZMA* and *GZMB*), cytokines (e.g., *IFNG*), death receptor ligands (e.g., *PRF1*), and immune effector molecules (e.g., *FASLG*). These findings suggest that CTL subtypes in metastatic sites undergo sustained immune activation, leading to enhanced cytotoxic functionality, a role predominantly driven by DNAJB1⁺ CTL. Although CTL populations overall exhibited increased cytotoxicity, as indicated by elevated *CD8 A* and *CD8B* expression, they also showed signs of immune escape and functional exhaustion, with the downregulation of *CTLA4*, *HAVCR2*, *TOX*, and *FASLG* suggesting that CTLs remain actively cytotoxic (Fig. 3I). Additionally, we performed pseudotime trajectory analysis on CTLs from the primary lesion and visualized gene expression changes throughout their developmental progression (Fig. S3B). Interestingly, we observed a pattern similar to that in metastatic CTLs, where cytotoxicity increased over time with the upregulation of *GZMA* and *CD8B*, while markers associated with immune evasion and exhaustion, including *LAG3*, *GNLY*, and *TBX21*, showed a declining trend. This similarity suggests that CTLs in both primary and metastatic lesions may undergo comparable developmental trajectories, following a shared functional and phenotypic evolution (Fig. S3C).

To explore the functional similarities and specificities of CTLs across different metastatic sites, we analyzed six shared pathways derived from differentially expressed genes in these metastases (Fig. S3D). Notably, we identified six commonly activated pathways across the four metastatic sites, with their functions primarily linked to neural system development (Fig. 3J, K). In site-specific contexts, CTLs in brain metastases exhibited activation of the EMT pathway, which may facilitate immune evasion within the tumor microenvironment (Fig. 3L). In contrast, lymph node metastases were characterized by highly active protein synthesis, reflecting the metabolic demands of CTLs in this environment (Fig. 3M). Bone metastases CTLs potentially play a role in suppressing viral replication and propagation, whereas CTLs in adrenal metastases demonstrated robust engagement with interferon response pathways, possibly contributing to an active defense against tumor growth and metastasis (Fig. 3N, O).

Further dissection of subtype-specific functions revealed that DNAJB1⁺ CTLs play a crucial role in cell regulation, apoptosis, and differentiation, involving TGF-beta signaling, apoptosis, and the P53 pathway. In contrast, CCL4⁺ CTLs are associated with cell proliferation, survival, and immune response, responding to IL6/JAK/STAT3 signaling and KRAS signaling. Additionally, GZMB⁺ CTLs are linked to cell growth, differentiation, and immune regulation, responding to TGF-beta, Hedgehog, IL6/JAK/STAT3, IL2/STAT5, and KRAS signaling. Meanwhile, MT1X⁺ CTLs primarily contribute to cell survival, growth, and metabolism, responding to TGF-beta signaling and PI3 K/AKT/mTOR signaling. Lastly, PLCG2⁺ CTLs are involved in cell differentiation, secretion, and survival, responding to Notch signaling, PI3 K/AKT/mTOR signaling, and protein secretion pathways (Fig. 3P). Together, these subtypes collectively contribute to the diverse immunological niches within lung cancer metastases, each executing specialized functions to shape the metastatic immune microenvironment.

### B cells and their subtypes play diverse roles in the niches of lung cancer metastases

**Fig. 4:**
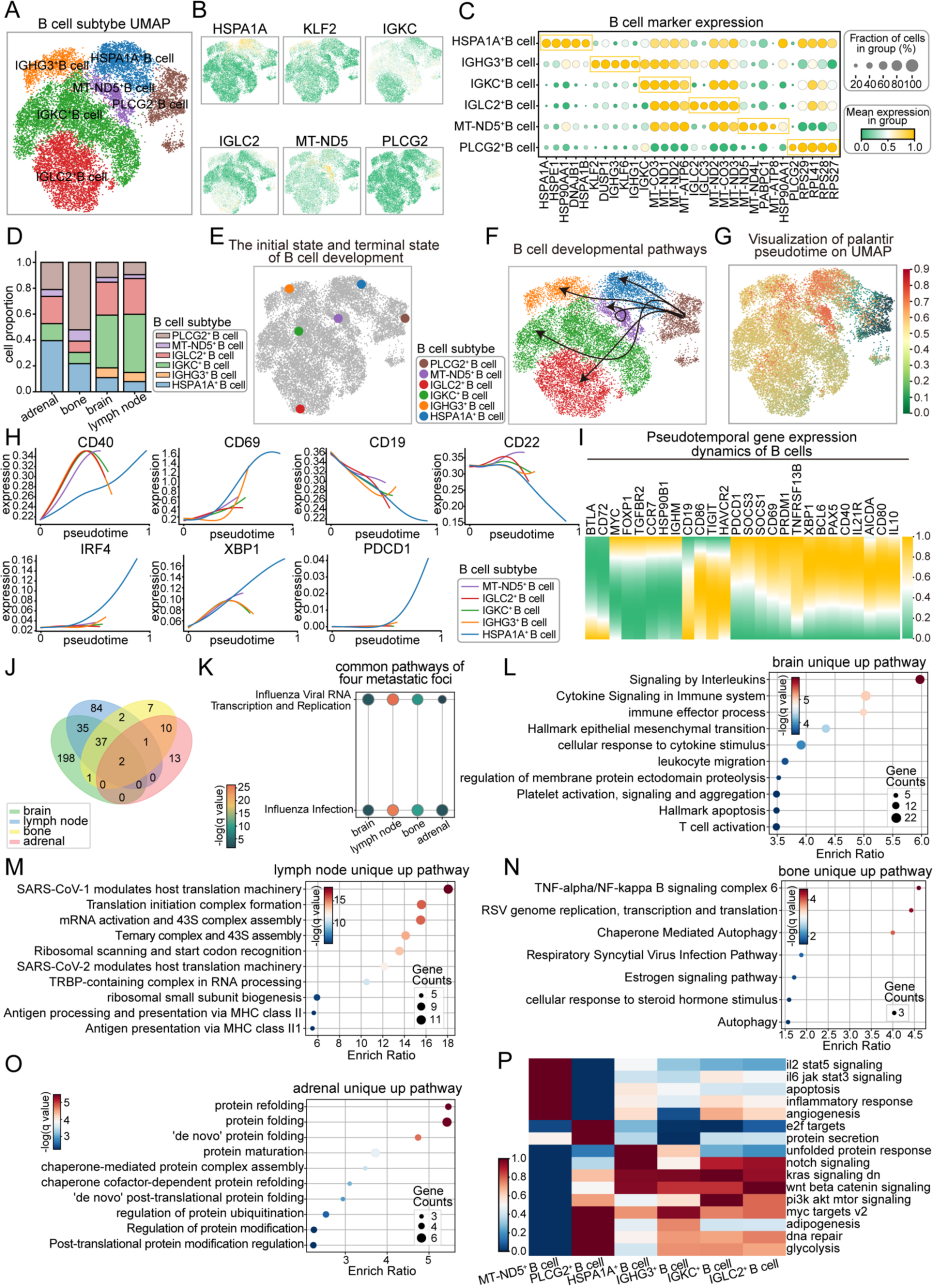
Characteristics, developmental trajectories, and gene expression analysis of B cell subtypes in metastatic sites. **A** UMAP clustering of B cell subtypes, showing the distribution of different subtypes. **B** Expression of key marker genes mapped onto the UMAP space. **C** Expression levels and distribution proportions of B cell marker genes; dot size indicates the fraction of cells in the group, and color represents gene expression levels. **D** Proportions of different B cell subtypes in various metastatic sites (adrenal, brain, bone, and lymph nodes). **E** Visualization of the initial and terminal states of B cell development in space. **F** Pseudotime analysis of B cell developmental trajectories, illustrating dynamic changes among subtypes during development. **G** Pseudotime trajectories visualized in UMAP space using the palantir algorithm. **H** Expression trends of key marker genes (e.g., *CD40*, *CD69*, *CD19*, *CD22*, *IRF4*, *XBP1*, and *PDCD1*) across pseudotime. **I** Heatmap showing the dynamic gene expression patterns of B cells over pseudotime, with different genes displaying temporal expression trends. **J** Venn diagram of pathways enriched for highly expressed genes in metastatic sites, illustrating shared and specific pathways among B cells in the brain, lymph node, bone, and adrenal sites. **K** Key pathways commonly enriched across the four metastatic sites in B cells. **L–O** Pathways were specifically enriched in B cells within each metastatic site (brain, lymph node, bone, and adrenal). **P** Heatmap of ssGSEA pathway scores for B cell subtypes, demonstrating activity differences across various signaling pathways

B cells constitute a significant cornerstone of the immune defense system. Through reclustering analysis, we identified six distinct B cell subtypes, with their highly expressed biomarkers color-coded for visualization (Figs. 4A–C, S4A). In adrenal metastases, HSPA1 A⁺ B cells dominated the landscape, whereas PLCG2⁺ B cells were most prevalent in bone metastases. Conversely, IGKC⁺ B cells represented the largest proportion in brain and lymph node metastases (Fig. 4D). Similarly, we designated PLCG2⁺ B cells as the developmental starting point due to their early activation and critical role in initiating immune responses [17, 18], and examined B cell functionality across developmental trajectories and transition processes within metastases (Fig. 4E–G). Overall, B cells displayed a relatively synchronized functional pattern. Their developmental and signaling capabilities gradually declined, as indicated by reduced *CD19* and *CD22* expression. In contrast, differentiation and antibody secretion functions were progressively activated, accompanied by enhanced immune checkpoint regulation, as evidenced by the upregulation of genes such as *CD40*, *CD69*, *IRF4*, and *XBP1* (Fig. 4H, I). Notably, the HSPA1 A⁺ B cell subtype persisted throughout the entire developmental process, consistently orchestrating these critical functions. However, we also performed pseudotime analysis on B cells in the primary lesion. In contrast, *CD19* and *CD22* expression increased, while *CD40*, *CD69*, *IRF4*, and *XBP1* expression decreased (Fig. S4B, C). This may suggest that B cells in the primary tumor microenvironment tend to remain in an immature or early activation state rather than progressing toward terminal differentiation. During the metastatic process, they may undergo functional reprogramming, transitioning toward terminal differentiation or acquiring immunoregulatory properties in response to the metastatic niche.

To further investigate the active biological processes and functional specializations of each subtype, we first performed differential gene expression analysis for each metastatic site (Fig. S4D), followed by pathway enrichment analysis. Interestingly, B cells across all metastatic sites commonly responded to viral infection pathways (Fig. 4J, K). Specifically, B cells played pivotal roles in immune responses to influenza virus infection, including antibody production and immune memory formation. However, the functional differences among metastatic sites were pronounced. For example, B cells in brain metastases prioritized intercellular communication and immune regulation, highlighted by pathways such as"Signaling by Interleukins"and"Cytokine Signaling in the Immune System"(Fig. 4L). In contrast, B cells in lymph node metastases were more actively engaged in immune responses against viral infections, while also contributing significantly to protein synthesis and translational regulation (Fig. 4M). On the other hand, B cells in bone metastases not only participated in immune regulation and inflammatory responses but also demonstrated unique antiviral capabilities (Fig. 4N). Meanwhile, B cells in adrenal metastases concentrated on protein folding processes, ensuring intracellular homeostasis and functional integrity (Fig. 4O). Subtype-specific functional analyses revealed distinct roles for each B cell subtype. MT-ND5⁺ B cells primarily contributed to inflammation and immune regulation. IGKC⁺ B cells excelled in the regulation of cell proliferation and survival. IGHG3⁺ and PLCG2⁺ B cells were pivotal in cell cycle control and DNA damage repair, whereas HSPA1 A⁺ B cells specialized in responding to endoplasmic reticulum stress and regulating cell fate. Finally, IGLC2⁺ B cells were actively involved in cell proliferation, differentiation, and migration (Fig. 4P). These specialized functions not only highlight the intricate division of labor among B cell subtypes but also underscore their collective contributions to the diverse immune microenvironment within lung cancer metastases.

### Functional dynamics and specialization of myeloid cells in lung cancer metastases

**Fig. 5:**
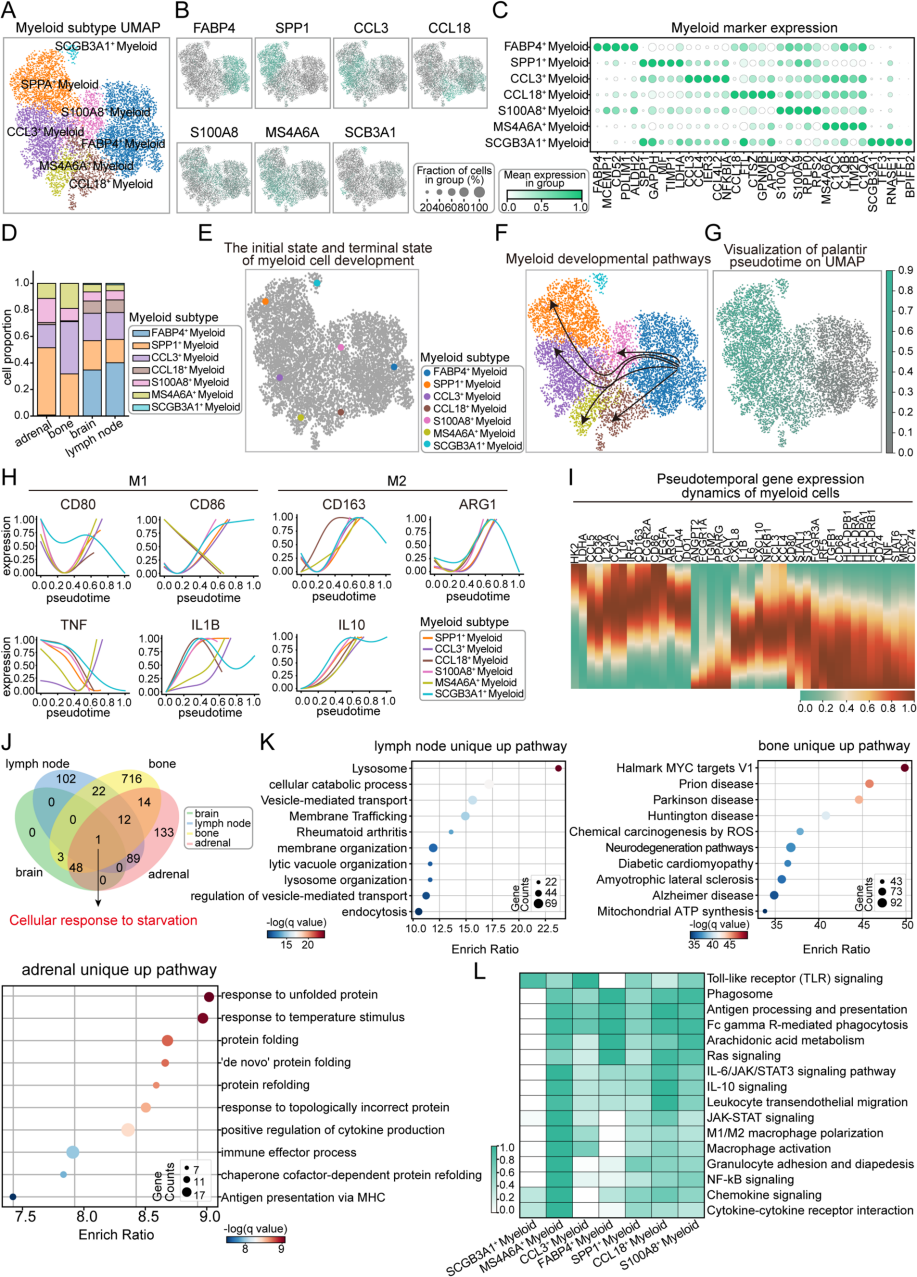
Developmental trajectories and heterogeneity of myeloid cells across four metastatic sites. **A** UMAP plot showing the distribution of myeloid cell subtypes, with different colors representing distinct myeloid cell subpopulations. **B** The expression of typical marker genes such as *FABP4*, *SPP1*, *CCL3*, etc. in the subpopulation of myeloid cells. **C** Dot plot showing the expression levels of highly expressed marker genes in myeloid cells. **D** Bar chart showing the proportions of different myeloid cell subtypes. **E** Schematic representation of the initial and terminal states of myeloid cell development, with the FABP4^+^Myeloid subtype defined as the developmental starting point. **F** UMAP plot illustrating the developmental trajectory of myeloid cell subtypes from the initial to the terminal state. **G** Visualization of initial pseudotime on UMAP, with different colors representing the various stages of cell development. **H** Pseudotime expression dynamics of genes associated with M1 and M2 states, showing trends in gene expression as myeloid cells transition between these two states. **I** Heatmap showing the pseudotime gene expression dynamics in myeloid cells, with color intensity representing gene expression levels. **J** Venn diagram showing shared and unique upregulated pathways across different metastatic sites (brain, lymph nodes, bone, and adrenal glands), with the only common upregulated pathway being the intercellular starvation pathway. **K** Enrichment analysis of unique upregulated pathways in lung cancer metastases in lymph nodes, bone, and adrenal glands. **L** Heatmap showing pathway ssGSEA scores in myeloid cell subtypes, with darker colors at higher scores

Myeloid cells, as the first line of defense against infections, exhibit remarkable functional diversity in lung cancer metastases. Through analysis, we identified seven myeloid cell subgroups and classified them based on the high expression of characteristic genes (Fig. 5A). UMAP and dot plots further displayed the characteristic markers and relative abundance of each myeloid cell subtype (Figs. 5B, C, S5A). Notably, SPP1⁺ myeloid cells were most abundant in adrenal metastases, whereas CCL3⁺ myeloid cells dominated in bone metastases. Meanwhile, FABP4⁺ myeloid cells accounted for a significant proportion in both brain and lymph node metastases (Fig. 5D). Using FABP4⁺ myeloid cells as the starting point for pseudotime analysis (Fig. 5E), due to their early activation and critical role in initiating immune responses, we traced their developmental trajectory into other subtypes over time (Fig. 5F, G) and inferred the functional changes accompanying differentiation [19, 20]. Overall, myeloid cell function gradually declined, including reduced immunosuppressive capacity, weakened anti-inflammatory properties, and a transition from an active state to exhaustion or terminal differentiation (Fig. S5B). However, M1-related genes exhibited disordered expression trends across all metastatic sites, reflecting the complex regulation of myeloid cells in the tumor microenvironment—characterized by both heterogeneity and competitive signaling dynamics. In contrast, M2-related genes showed a consistent upward trend, reflecting the tumor microenvironment’s integrative reprogramming of myeloid cells toward immunosuppressive functions (Fig. 5H, I). In the primary lesion, the developmental trajectory of myeloid cells exhibits the opposite pattern, characterized by enhanced immunosuppressive and metabolic adaptation. This is reflected in the upregulation of *TGFB1*, *PPARG*, *STAT6*, *MRC1*, and *CD74*, indicating a shift toward an anti-inflammatory and immunosuppressive phenotype. Additionally, *ACLY* and *TGM2* upregulation suggests increased metabolic adaptation, while the downregulation of *CCL2*, *CCL3*, and *VEGFA* indicates a reduction in chemotactic and pro-inflammatory capacity. These changes may contribute to shaping an immunosuppressive microenvironment conducive to tumor growth, reducing anti-tumor immune pressure, and promoting tumor survival and dissemination (Fig. S5C, D).

We found that myeloid cells across all metastatic sites responded to cellular starvation signals, demonstrating a universal adaptation to metabolic changes. Interestingly, myeloid cells in brain metastases lacked uniquely upregulated genes, suggesting their functions were more aligned with basic immune activities (Figs. 5J, S5E). In terms of site-specific pathway responses, myeloid cells in lymph node metastases exhibited enhanced lysosomal function, while those in bone metastases showed upregulation of *MYC* target genes. In adrenal metastases, myeloid cells focused on maintaining protein homeostasis and adapting to temperature fluctuations (Fig. 5K). Notably, regardless of the proportional differences in myeloid subtypes across metastatic sites, their functions demonstrated both commonalities and remarkable specificity. In immune regulation, myeloid cells showed functional consistency, such as the activation of Toll-like receptor signaling and antigen processing and presentation pathways. However, distinct subtypes exhibited clear functional specialization, for example MS4 A6 A⁺ myeloid cells were particularly active in chemokine signaling and cytokine-receptor interactions, while CCL18⁺ myeloid cells dominated in the regulation of inflammatory cytokine signaling pathways (Fig. 5L).

### Functional heterogeneity of CAFs and their adaptive roles in metastatic sites

**Fig. 6:**
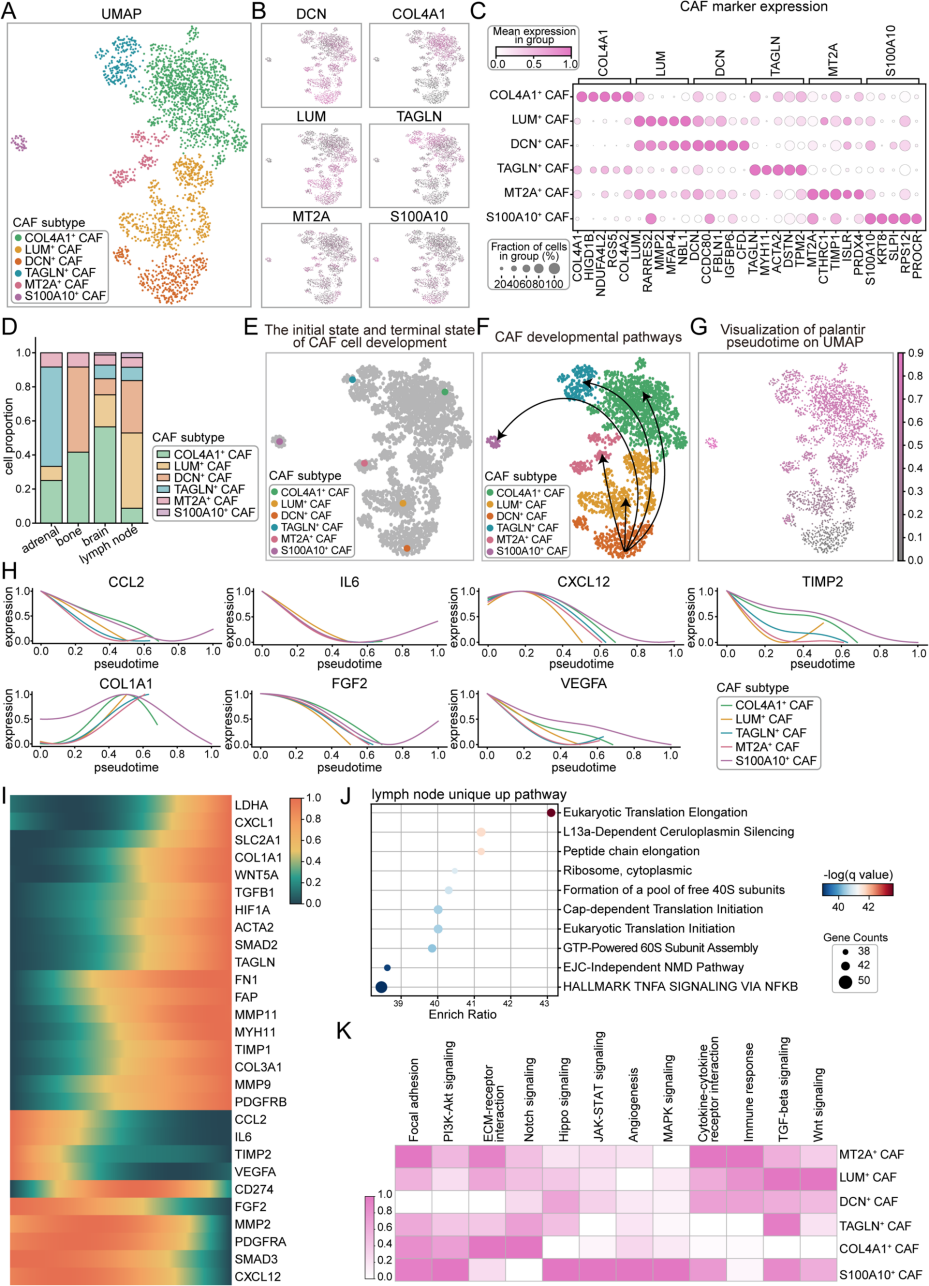
Comprehensive analysis of CAFs heterogeneity, developmental trajectories, and genes expression dynamics across metastatic sites. **A** UMAP plot illustrating CAF subtypes based on scRNA-seq data, with distinct colors representing different CAF subgroups. **B** Expression levels of *DCN*, *COL4 A1*, *LUM*, *TAGLN*, *MT2 A*, and *S100 A10* genes across various CAF subtypes. **C** Expression levels of CAF marker genes, including *COL4 A1*, *LUM*, *TAGLN*, *MT2 A*, and *S100 A10*, in different CAF subtypes. **D** Bar chart showing the proportions of CAF subtypes across adrenal gland, bone, brain, and lymph node metastatic sites. **E** UMAP plot identifies the starting and terminal states of CAFs development, with DCN^+^ CAFs defined as the developmental starting point. **F** Developmental trajectories observed across different CAF subtypes. **G** Visualization of CAFs developmental pathways on a UMAP plot, where pink shades indicate cells closer to the terminal developmental state. **H** Pseudotime expression profiles of *CCL2*, *IL6*, *CXCL12*, *TIMP2*, *COL1 A1*, *FGF2* and *VEGFA* genes in various CAF subtypes. **I** Pseudotime expression dynamics of all genes within CAFs. **J** Enriched pathways of highly expressed genes in lymph node metastases. **K** ssGSEA pathway activity scores across CAF subtypes, with deeper colors representing higher scores

CAFs play a pivotal role in modulating the tumor microenvironment. The classification of CAF subtypes was performed based on the highly expressed markers within each subgroup, through which we identified six distinct CAF subgroups (Figs. 6A–C, S6A), each exhibiting specific distribution patterns across different metastatic sites: TAGLN⁺ CAFs dominated the adrenal gland, *DCN*⁺ CAFs were prevalent in bone metastases, COL4 A1⁺ CAFs predominated in the brain, while LUM⁺ CAFs were highly enriched in lymph node metastases (Fig. 6D). Further investigation of the pseudo-time trajectory of DCN⁺ CAFs (Fig. 6E–G) revealed dynamic functional changes, and designating DCN⁺ CAFs as the developmental starting point allowed us to capture the early activation and differentiation processes of these cells, which play a critical role in ECM remodeling and immune modulation within the tumor microenvironment [21, 22]. As pseudo-time progressed, the expression levels of *COL1 A1*, *WNT5 A*, and *HIF1 A* increased, suggesting that CAFs play crucial roles in extracellular matrix remodeling, developmental pathway regulation (e.g., WNT signaling), and adaptation to hypoxic conditions, collectively supporting tumor growth and invasion. In contrast, the expression of another set of genes, including *CCL2*, *IL6*, *CXCL12*, *TIMP2*, *FGF2*, *VEGFA*, and *SMAD3*, showed a declining trend, indicating a gradual attenuation of CAF-mediated functions in immune modulation, inflammatory response, angiogenesis, and matrix-related signaling pathways (Figs. 6H, I, S6B). In the primary lesion, CAF promotes local tumor growth and dissemination by upregulating genes such as *FGF2, IL6, MMP2, SMAD3, CXCL2,* and *CCL2*, while simultaneously downregulating *HIF1 A*, *COL1 A1*, and *VEGFA* (Fig. S6C, D). Notably, although both primary and metastatic CAFs play a role in stromal regulation, their functions differ. Primary tumor CAFs primarily create the initial conditions for metastasis, whereas metastatic CAFs support the survival and adaptation of distant metastatic tumors through stromal remodeling, hypoxia adaptation, and WNT signaling. This phenomenon suggests a “two-stage” role of CAFs in tumor progression: primary CAFs facilitate metastasis, while metastatic CAFs provide sustained support for tumor adaptation and survival in the new microenvironment.

Pathway enrichment analysis of CAF-specific highly expressed genes in metastatic lesions unveiled an additional dimension to CAF functionality. Notably, CAFs in lymph node metastases exhibited characteristics associated with enhanced protein synthesis and cell proliferation, suggesting their potential involvement in promoting metabolic activity and tumor cell proliferation (Figs. S6E, 6J). Furthermore, each CAF subgroup demonstrated functional specificity. For example, MT2 A⁺ CAFs excelled in regulating cell migration and immune-related processes, while LUM⁺ CAFs were predominantly involved in EMT and tumor cell differentiation, potentially driving tumor dissemination. TAGLN⁺ CAFs contributed to extracellular matrix remodeling and tumor cell invasion through TGF-β signaling pathways. S100 A10⁺ CAFs displayed multifaceted roles in cell proliferation, migration, and angiogenesis, whereas COL4 A1⁺ CAFs primarily provided structural support and regulated signaling within the tumor microenvironment (Fig. 6K).

### Heterogeneity and site-specific functions of tumor cells in lung cancer metastases

**Fig. 7:**
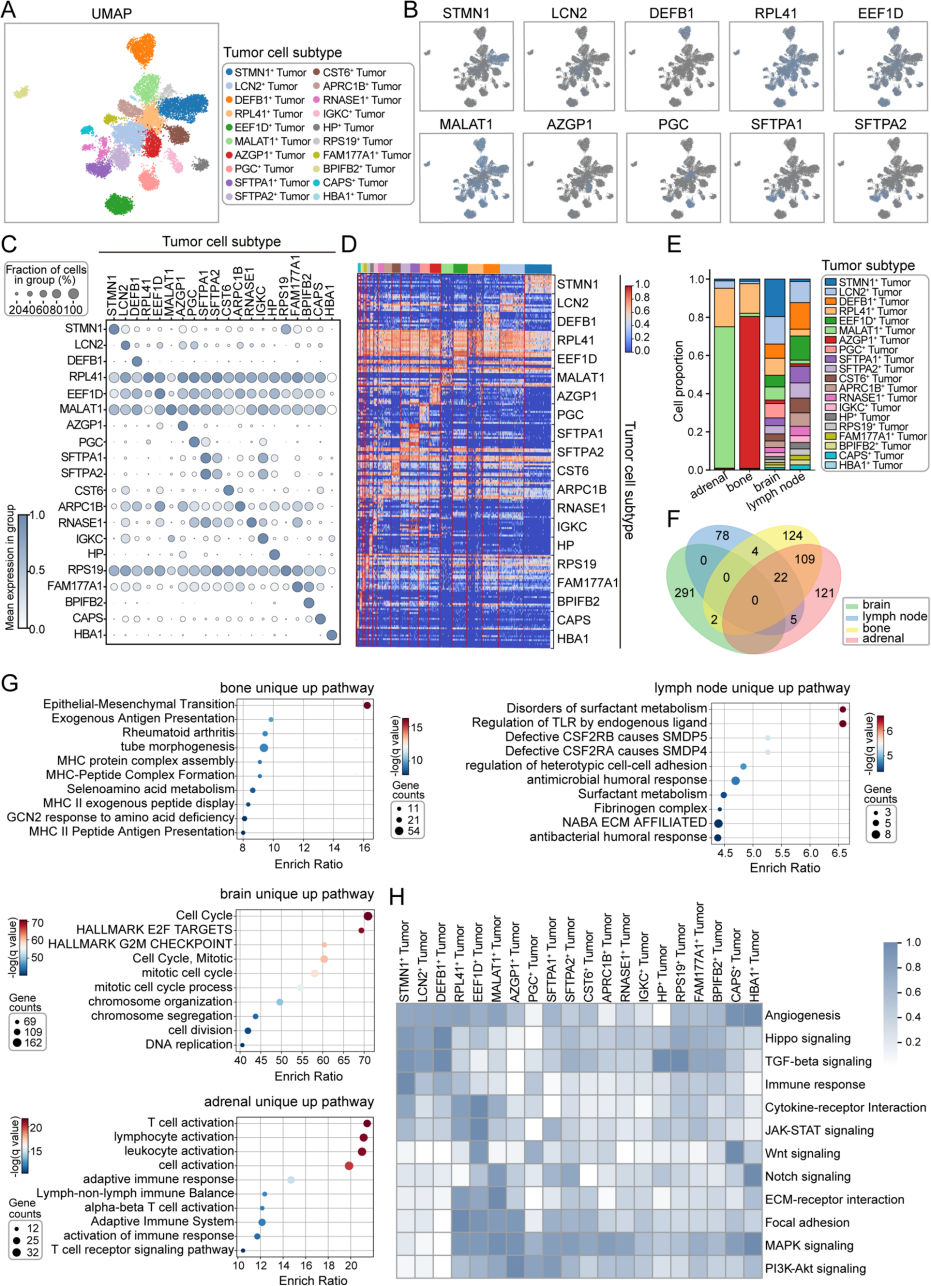
Characterization of tumor cell subtypes and pathway signatures in metastatic lung cancer.** A** UMAP plot illustrating tumor cell subtypes, with distinct colors representing different subtypes. **B** Expression distribution of key marker genes (e.g., *STMN1*, *LCN2*, *DEFB1*) across tumor subtypes. **C** Dot plot summarizing the average expression levels and proportion of cells expressing marker genes in each tumor subtype. **D** Heatmap showing gene expression profiles across tumor subtypes. **E** Proportions of tumor subtypes across four metastatic sites of lung cancer (adrenal, bone, brain, lymph node). **F** Venn diagram comparing pathways enriched in highly expressed genes across the four metastatic sites, with no shared pathways identified. **G** Enrichment analysis of uniquely upregulated pathways in tumor cells from each metastatic site. **H** Heatmap displaying ssGSEA pathway scores for each tumor subtype, with darker colors indicating higher scores

The heterogeneity of tumor cells in lung cancer metastases has been meticulously delineated in our analysis, which identified 20 distinct cell clusters (Fig. 7A). Their unique characteristics were visualized through feature maps and heatmaps, highlighting the genes with the greatest variability within each cluster (Figs. 7B, C, S7A). These clusters exhibited co-expression patterns of gene modules linked to critical biological processes, including cell cycle regulation (e.g., *STMN1* and *MALAT1*), immune response (e.g., *LCN2* and *DEFB1*), and protein synthesis and processing (e.g., *RPL41*, *EEF1D*, and *ARPC1B*) (Fig. 7D). Notably, MALAT1⁺ tumor cells predominantly localized in adrenal metastases, while AZGP1⁺ tumor cells were enriched in bone metastases. Similarly, STMN1⁺ tumor cells were prominent in brain metastases, whereas DEFB1⁺ tumor cells were more abundant in lymph node metastases (Fig. 7E).

Intriguingly, pathway enrichment analyses of the genes highly expressed in each metastatic site revealed almost entirely non-overlapping signaling pathways (Figs. S7B, 7F). For instance, tumor cells in bone marrow metastases exhibited marked activation of the EMT pathway, a transformation closely associated with increased tumor invasiveness, enhanced metastatic potential, and greater drug resistance. In contrast, tumor cells in lymph nodes appeared to modulate pulmonary surfactant metabolism and endogenous ligand-driven Toll-like receptor signaling, potentially facilitating immune evasion. In brain metastases, tumor cells predominantly regulate the cell cycle, supporting rapid proliferation. Meanwhile, in adrenal metastases, tumor cells orchestrated a complex immune regulatory network by activating T cells, lymphocytes, and leukocytes (Fig. 7G). Further functional analysis of these tumor cell subclusters revealed their distinct roles across diverse signaling pathways. For example, STMN1⁺ tumor cells displayed heightened activity in immune response pathways, acting as pivotal “mediators” of immune modulation within metastatic niches. The association of DEFB1⁺ tumor cells with the Hippo signaling pathway suggested their involvement in regulating cell proliferation, tumor suppression, and tissue regeneration. Additionally, the activation of the TGF-beta signaling pathway in DEFB1⁺ tumor cells indicated a role in modulating cell proliferation, differentiation, apoptosis, and immune regulation, underscoring their contribution to shaping the metastatic microenvironment. Lastly, MALAT1⁺ tumor cells exhibited significant activity in the ECM-receptor interaction pathway, implying roles in enhancing cell adhesion, migration, and signal transduction efficiency (Fig. 7H).

### Immune-tumor communication shapes metastatic niches and reveals therapeutic targets

**Fig. 8:**
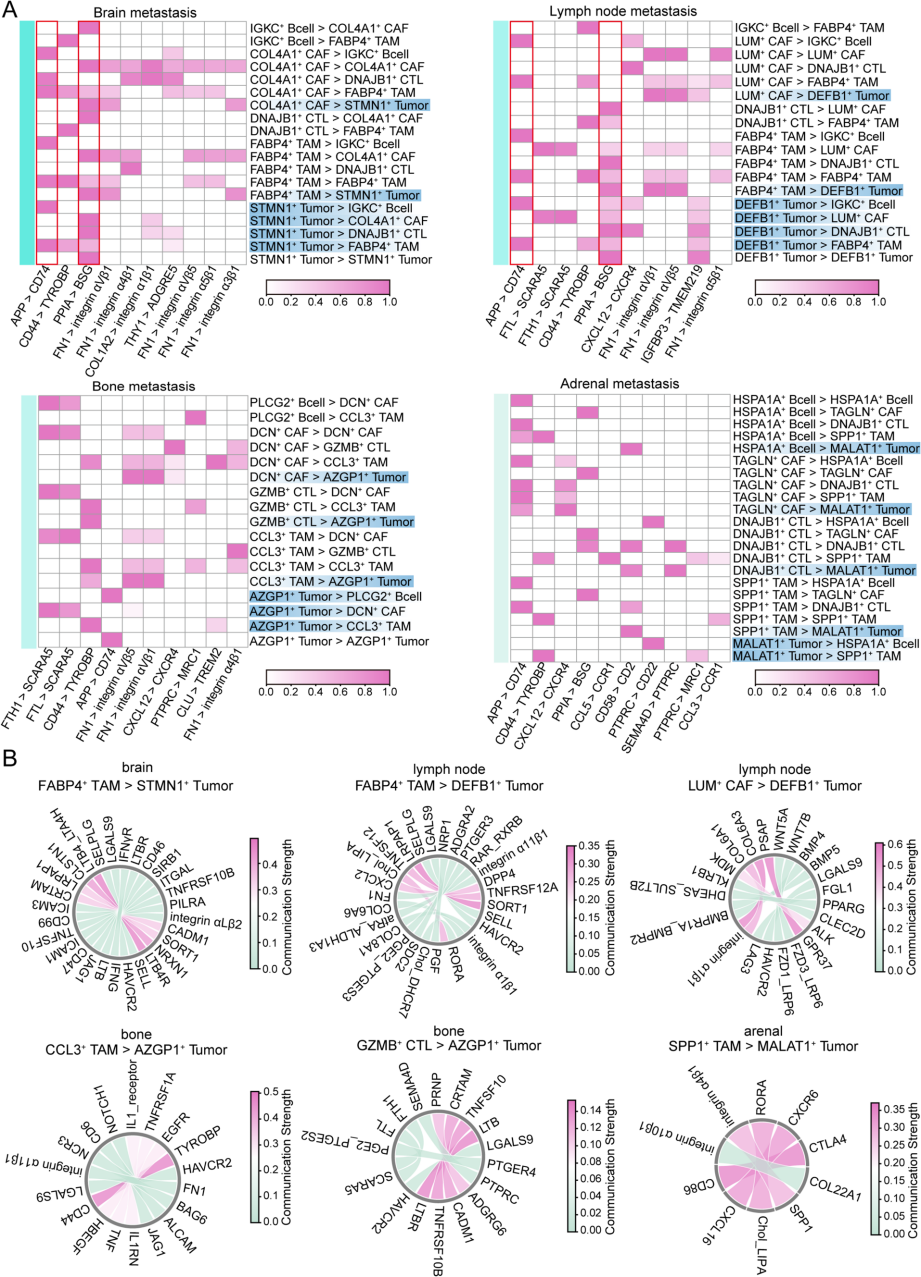
Ligand-receptor interactions across metastatic sites and cell-type-specific communication patterns.** A** Heatmaps depicting the ligand-receptor interaction pairs identified in brain, lymph node, bone, and adrenal metastatic sites of lung cancer. The interactions are categorized by cell types, with significant pairs highlighted. The color gradient represents the communication strength, ranging from low (white) to high (pink). **B** Circular plots illustrating selected ligand-receptor interactions between key cell types in specific metastatic sites. Each plot highlights the communication strength of ligand-receptor pairs, with arrows indicating directional interactions between sender and receiver cell types. Specific interactions, such as FABP4⁺ TAM to STMN1⁺ tumor cells in the brain and SPP1⁺ TAM to MALAT1⁺ tumor cells in the adrenal gland, are emphasized

To investigate how immune cells and tumor cells communicate to shape the unique niches of each metastatic site and to identify potential therapeutic targets, we analyzed cell–cell communication between the most abundant immune cell and tumor cell subtypes in each metastatic site, focusing on the top 10 receptor-ligand pairs ranked by communication strength. The analysis revealed that specific subtypes of B cells, CAFs, TAMs, and CTLs were the predominant immune cells regulating tumor cells across all metastatic sites (Fig. 8A). In brain metastases, COL4 A1⁺ CAFs and FABP4⁺ TAMs strongly regulated STMN1⁺ tumor cells via PPIA-BSG, while similar communication patterns were observed in lymph node metastases, with LUM⁺ CAFs and FABP4⁺ TAMs regulating DEFB1⁺ tumor cells through FN1-Integrin and PPIA-BSG. In both sites, APP-CD74 served as a key receptor-ligand axis. In bone metastases, tumor regulation was mediated by CAF and TAM via FN1-Integrin, and by CTLs through CD44-TYROBP. Adrenal metastases exhibited more site-specific interactions, with APP-CD74 remaining a critical communication axis.

Analysis of immune checkpoint expression revealed universally high expression levels across all cell types in adrenal metastases, which was particularly striking. In bone metastases, however, *CTLA4* expression was detected exclusively on B cells, suggesting a unique functional role in this metastatic niche (Fig. S8). Building on these findings, we further analyzed immune checkpoint-related communication between the most abundant cell subtypes. Although tumor cells exhibited weak interactions with other cells through the *HAVCR2*-*LGALS9* axis, a pronounced and robust interaction was observed between myeloid cells and tumor cells in adrenal metastases via the CTLA4-CD86 pathway (Fig. 8B).

## Discussion

Current studies primarily focus on the immune microenvironment of primary lung cancer or individual metastatic sites, failing to comprehensively reveal the complex heterogeneity between metastatic sites [23, 24]. This work is the first to integrate and systematically analyze scRNA-seq data covering four major metastatic sites of lung cancer, including the brain, lymph nodes, bone, and adrenal gland. Through this approach, we generated a comprehensive atlas of the cellular composition, transcriptional landscapes, developmental trajectories, and functional divergence of pivotal cellular populations across distinct metastatic niches. Building on this foundation, we conducted an innovative and in-depth investigation into the functional heterogeneity of immune and tumor cells, the dynamic architecture of intercellular communication networks, the site-specific molecular signatures of diverse metastatic lesions, and the metastatic niche-specific heterogeneity of immune checkpoints. Through these analyses, we sought to characterize the adaptive changes in the tumor microenvironment across metastatic sites and explore potential mechanisms contributing to immune evasion.

In T cells, the proportion of T cells is high across all metastatic sites, particularly in CTL cells, which exhibit exceptional immune regulatory functions. Although CTL cells gradually enhance cytotoxicity in each metastatic site, they also show trends of immune evasion and functional exhaustion. Notably, the proportion of exhausted CTLs is particularly high in the adrenal gland metastatic site, seemingly a"compromise"after a long-term battle between the tumor microenvironment and immune defense. Interestingly, B cells in all metastatic sites respond to viral infection pathways, especially in the lymph node, an immune center, where tumor cells may induce B cells to respond to viral infection pathways, thereby suppressing immune attack and helping the tumor evade immune surveillance. This may be because the majority of lung cancer cases are closely related to smoking and viral infections [25–27]. In myeloid cells, we found significant fluctuation in the expression of M1-related genes across different metastatic sites, reflecting the complexity and dynamics of myeloid cell regulation in the tumor microenvironment. In contrast, the expression of M2-related genes generally increased, indicating a"reprogramming"process of myeloid cells in the tumor microenvironment, shifting from the initial pro-inflammatory M1 type to the M2 type, which is mainly involved in immune suppression and tissue repair functions [28, 29]. In particular, MS4 A6 A⁺ myeloid cells exhibit an active state in chemokine signaling and cytokine receptor interactions, while CCL18⁺ myeloid cells dominate the regulation of inflammatory cytokine signaling pathways. This division of labor illustrates how myeloid cells form a"basic immune defense"in metastatic sites to adapt to changes in the tumor microenvironment. The gene expression characteristics of tumor cells demonstrate the specificity of metastatic sites, with tumor cells in each metastatic site displaying unique"temperament and characteristics,"meaning they create distinct pathological ecological niches within their respective microenvironments. The pathways enriched in tumor cells from different metastatic sites do not overlap, further proving that their pathological features differ significantly. This phenomenon is similar to the behavior of CAFs. CAFs support the immune evasion of tumor cells by secreting chemokines, while tumor cells promote this process by activating CAFs or through metabolic reprogramming [30–32]. This intricate cell cooperation may be one of the reasons for the formation of therapeutic resistance barriers in various metastatic sites.

Furthermore, we conducted an in-depth analysis of cell communication and immune checkpoints between the most abundant immune cell subtypes and tumor cells in the four metastatic sites to explore potential therapeutic targets. As expected, we found that the regulatory relationship between CAFs and tumor cells was particularly significant in brain and lymph node metastases, with key ligands such as PPIA-BSG, FN1-Integrin, and APP-CD74 playing crucial roles in these metastases. For example, a study by Xingzhi Wang et al. showed that PPIA-BSG plays a key role in breast cancer lung metastasis [33]; meanwhile, Xuefeng Li et al. revealed that macrophages induce FN1-Integrinα5 signaling in tumor cells, increasing cancer cell drug resistance [34]. These findings not only highlight the importance of ligands on myeloid cells but also underscore their central role in immune evasion and resistance formation in lung cancer metastasis. Even more interestingly, scRNA-seq also revealed the role of the APP-CD74 signaling axis in testicular cancer, further confirming the potential immune regulatory role of this axis in the tumor microenvironment [35]. These data suggest that ligands on myeloid cells play a role in lung cancer metastasis that goes beyond a single immune evasion mechanism, involving a broad process of immune evasion and resistance formation [36, 37]. Additionally, we observed that immune checkpoint expression was particularly prominent in adrenal metastases, especially the CTLA4-CD86 receptor-ligand interaction, which was notably significant in the communication between SPP1⁺TAM and MALAT1⁺ tumor cells. It is worth noting that although previous studies have shown that blocking *CTLA4* can effectively overcome TKI resistance in lung cancer brain metastasis, there is limited research on immune targets in adrenal metastases, which may be related to their lower incidence [38].

Undoubtedly, while we have successfully integrated single-cell data from four major metastatic sites of lung cancer and conducted an in-depth analysis of the heterogeneity and functional differences of immune cells, there remain certain limitations. Our focus was primarily on the most abundant immune cell subtypes for cell–cell communication analysis, which did not fully consider the potential impact of other subtypes on the tumor microenvironment. Additionally, although some potential therapeutic targets have been identified, the current analysis is confined to transcriptomic data, and cannot reveal dynamic changes at the protein level or metabolic activities. Therefore, future studies need to further validate these targets through in vitro and in vivo experiments and deepen our understanding of the microenvironment of lung cancer metastasis, with the goal of translating these findings into clinical applications.

## Supplementary Information

Additional file1Batch correction effect visualization. A Batch-corrected UMAP plot of all samples. B Batch-corrected UMAP plot of the GSE123902, GSE131907, GSE148071, and GSE186344 datasets

Additional file2Expression of T cell genes and differential gene expression analysis in four metastatic sites. A UMAP plots depict the marker gene expression characteristics of T cell subpopulations. B Comparison of T cell-specific highly expressed genes in brain metastases, lymph node metastases, bone metastases, and adrenal metastases, relative to the other three metastatic sites. Red indicates genes with high expression in the specific metastasis, while blue represents genes with high expression in the other three metastases

Additional file3Expression of CTL subtypes marker genes and differential gene expression analysis in four metastatic sites. A UMAP visualization depicting the characteristic expression profiles of CTL subtypes marker genes. B UMAP visualization of CTLs from four metastatic sitesand pseudotime developmental trajectory using primary lesion CTLs as the starting point, with intermediate CTLs representing transitional CTL subtype. C Gene expression dynamics of CTLs from the primary lesion along pseudotime, with color intensity representing gene expression levels. D A comparative analysis of genes exhibiting elevated expression specifically in CTLs from metastatic sites including the brain, lymph node, bone, and adrenal glands, in contrast to the other three metastatic locations. Red indicates genes with high expression in the specific metastasis, while blue represents genes with high expression in the other three metastases

Additional file4UMAP visualization and differential gene expression analysis of B cell subtypes marker genes in metastatic sites. A UMAP visualization depicting the characteristic expression profiles of B cell subtypes marker genes. B UMAP visualization of B cells from four metastatic sitesand pseudotime developmental trajectory using primary lesion B cells as the starting point, with intermediate B cell representing transitional B cell subtype. C Gene expression dynamics of B cells from the primary lesion along pseudotime, with color intensity representing gene expression levels. D A comparative analysis of genes exhibiting elevated expression specifically in B cells from metastatic sites including the brain, lymph node, bone, and adrenal glands, in contrast to the other three metastatic locations. Red indicates genes with high expression in the specific metastasis, while blue represents genes with high expression in the other three metastases

Additional file5UMAP visualization, pseudotime analysis, and differential gene expression in myeloid cells across metastatic sites. A UMAP visualization of marker genes in myeloid cell subpopulations, with red indicating higher expression levels. B Pseudotime trajectory analysis showing the expression patterns of TGFB1, MRC1, TGM2, PPARG, VEGFR, CCL2, and STAT6 in myeloid cell subpopulations. C UMAP visualization of myeloid cells from four metastatic sitesand pseudotime developmental trajectory using primary lesion myeloid cells as the starting point, with intermediate myeloid cell representing transitional myeloid cell subtype. C Gene expression dynamics of myeloid cells from the primary lesion along pseudotime, with color intensity representing gene expression levels. D A comparative analysis of genes exhibiting elevated expression specifically in myeloid cells from metastatic sites including the brain, lymph node, bone, and adrenal glands, in contrast to the other three metastatic locations. Red indicates genes with high expression in the specific metastasis, while blue represents genes with high expression in the other three metastases

Additional file6UMAP visualization, pseudotime analysis, and differential gene expression in CAFs across metastatic sites. A UMAP visualization of marker genes in CAF subpopulations, with pink indicating higher expression levels. B Pseudotime trajectory analysis showing the expression patterns of WNT5 A, HIF1 A, and SMAD3 in CAF subpopulations. C UMAP visualization of CAFs from four metastatic sitesand pseudotime developmental trajectory using primary lesion CAFs as the starting point, with intermediate CAFs representing transitional CAF subtype. C Gene expression dynamics of CAFs from the primary lesion along pseudotime, with color intensity representing gene expression levels. D A comparative analysis of genes exhibiting elevated expression specifically in CAFs from metastatic sites including the brain, lymph node, bone, and adrenal glands, in contrast to the other three metastatic locations. Pink indicates genes with high expression in the specific metastasis, while grey represents genes with high expression in the other three metastases

Additional file7UMAP visualization and differential gene expression in tumor cells across metastatic sites. A UMAP visualization of marker genes in tumor cell subpopulations, with blue indicating higher expression levels. B A comparative analysis of genes exhibiting elevated expression specifically in tumor cells from metastatic sites including the brain, lymph node, bone, and adrenal glands, in contrast to the other three metastatic locations. Blue indicates genes with high expression in the specific metastasis, while grey represents genes with high expression in the other three metastases

Additional file8Expression of immune checkpoints across immune and tumor cells in all metastatic sites of lung cancer. A Expression of immune checkpoints on T cells across metastatic sites. B Expression of immune checkpoints on B cells. C Expression of immune checkpoints on CAFs. D Expression of immune checkpoints on myeloid cells. E Expression of immune checkpoints on tumor cells. *** Means P < 0.001; ** Means P < 0.01; * Means P < 0.05

## Data Availability

The datasets in this study can be found online, https://www.ncbi.nlm.nih.gov/geo/query/acc.cgi?acc=GSE123902, GSE123902. https://www.ncbi.nlm.nih.gov/geo/query/acc.cgi?acc=GSE131907, GSE131907. https://www.ncbi.nlm.nih.gov/geo/query/acc.cgi?acc=GSE148071, GSE148071. https://www.ncbi.nlm.nih.gov/geo/query/acc.cgi?acc=GSE186344, GSE186344
