# Supplementary figures and images for: Single-cell profiling uncovers the intricate pathological niche diversity in brain, lymph node, bone, and adrenal metastases of lung cancer
Source: Discov Oncol. 2025 Apr 10;16:512. doi: 10.1007/s12672-025-02269-w (PMC11985749; doi:10.1007/s12672-025-02269-w)

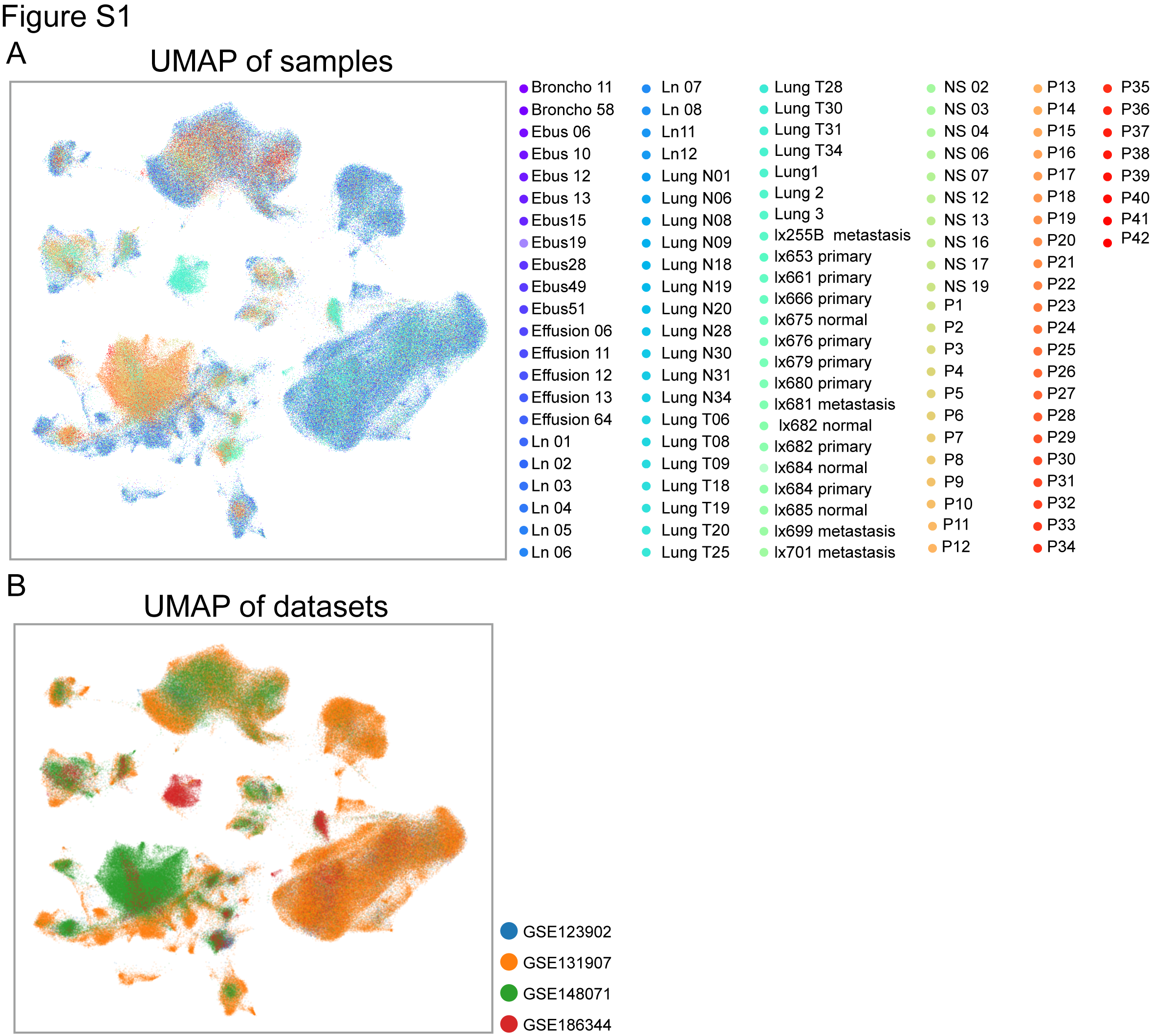

Supplement: Supplementary file 1 — Additional file1Batch correction effect visualization. A Batch-corrected UMAP plot of all samples. B Batch-corrected UMAP plot of the GSE123902, GSE131907, GSE148071, and GSE186344 datasets [file 12672_2025_2269_MOESM1_ESM.tif]

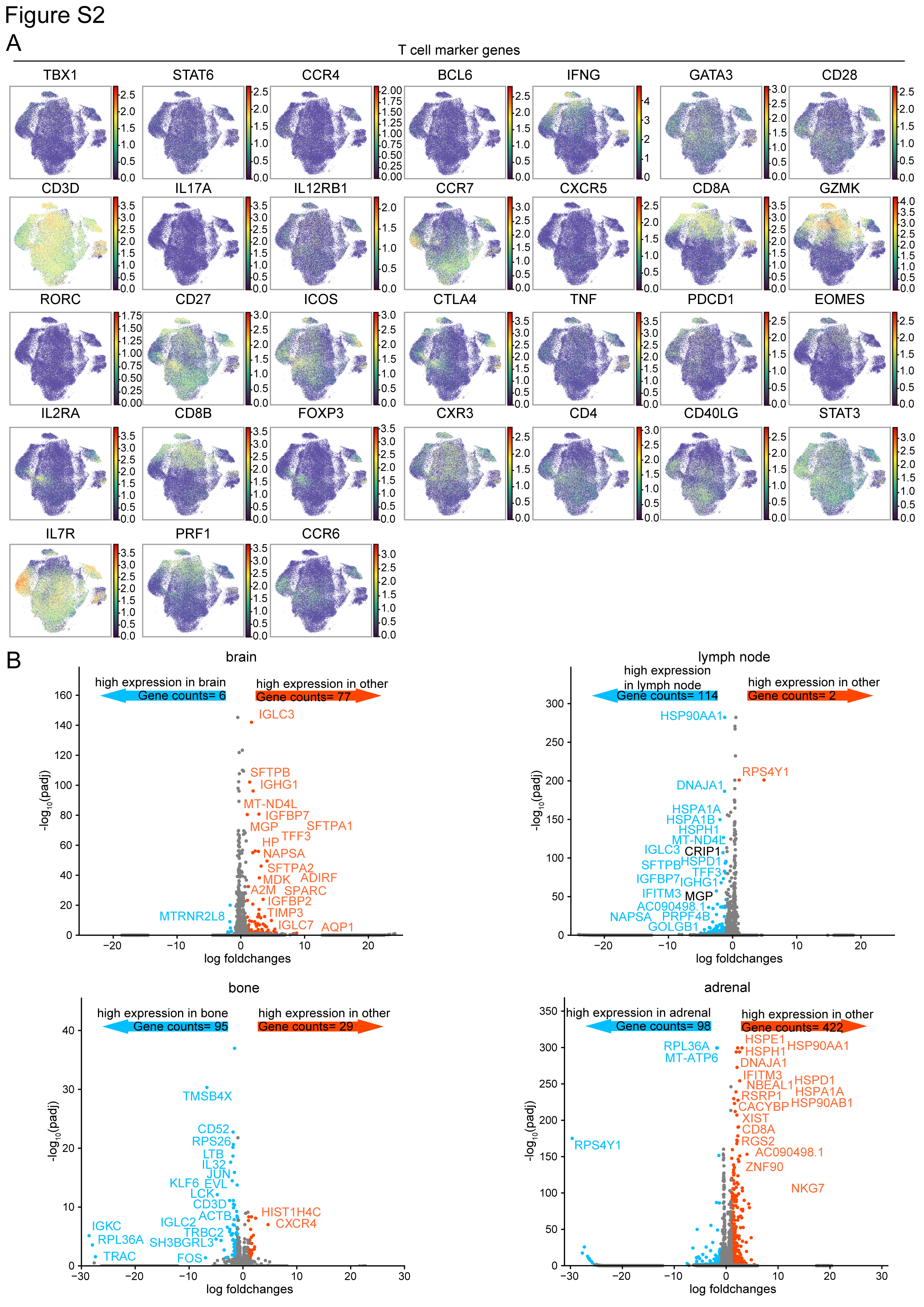

Supplement: Supplementary file 2 — Additional file2Expression of T cell genes and differential gene expression analysis in four metastatic sites. A UMAP plots depict the marker gene expression characteristics of T cell subpopulations. B Comparison of T cell-specific highly expressed genes in brain metastases, lymph node metastases, bone metastases, and adrenal metastases, relative to the other three metastatic sites. Red indicates genes with high expression in the specific metastasis, while blue represents genes with high expression in the other three metastases [file 12672_2025_2269_MOESM2_ESM.tif]

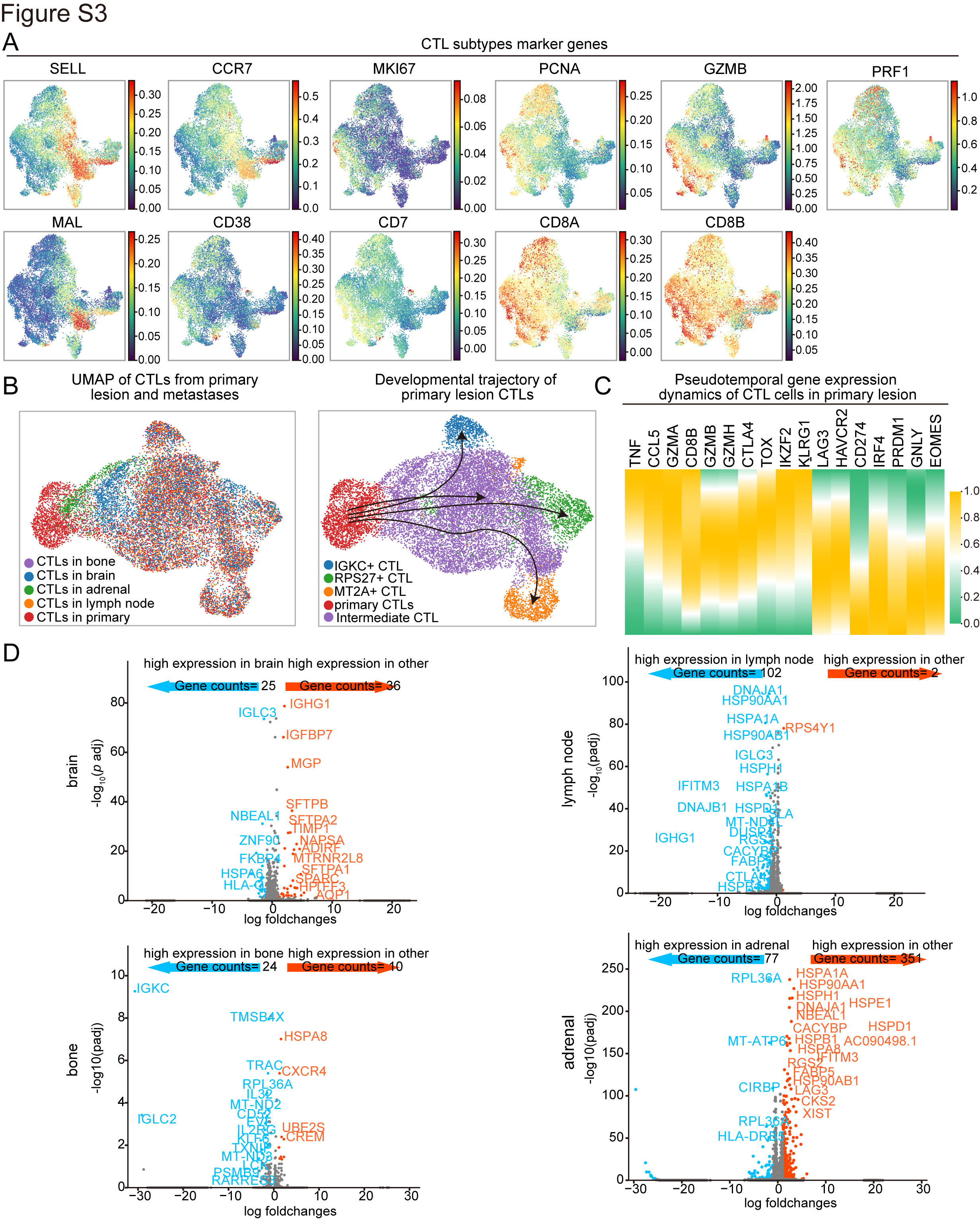

Supplement: Supplementary file 3 — Additional file3Expression of CTL subtypes marker genes and differential gene expression analysis in four metastatic sites. A UMAP visualization depicting the characteristic expression profiles of CTL subtypes marker genes. B UMAP visualization of CTLs from four metastatic sitesand pseudotime developmental trajectory using primary lesion CTLs as the starting point, with intermediate CTLs representing transitional CTL subtype. C Gene expression dynamics of CTLs from the primary lesion along pseudotime, with color intensity representing gene expression levels. D A comparative analysis of genes exhibiting elevated expression specifically in CTLs from metastatic sites including the brain, lymph node, bone, and adrenal glands, in contrast to the other three metastatic locations. Red indicates genes with high expression in the specific metastasis, while blue represents genes with high expression in the other three metastases [file 12672_2025_2269_MOESM3_ESM.tif]

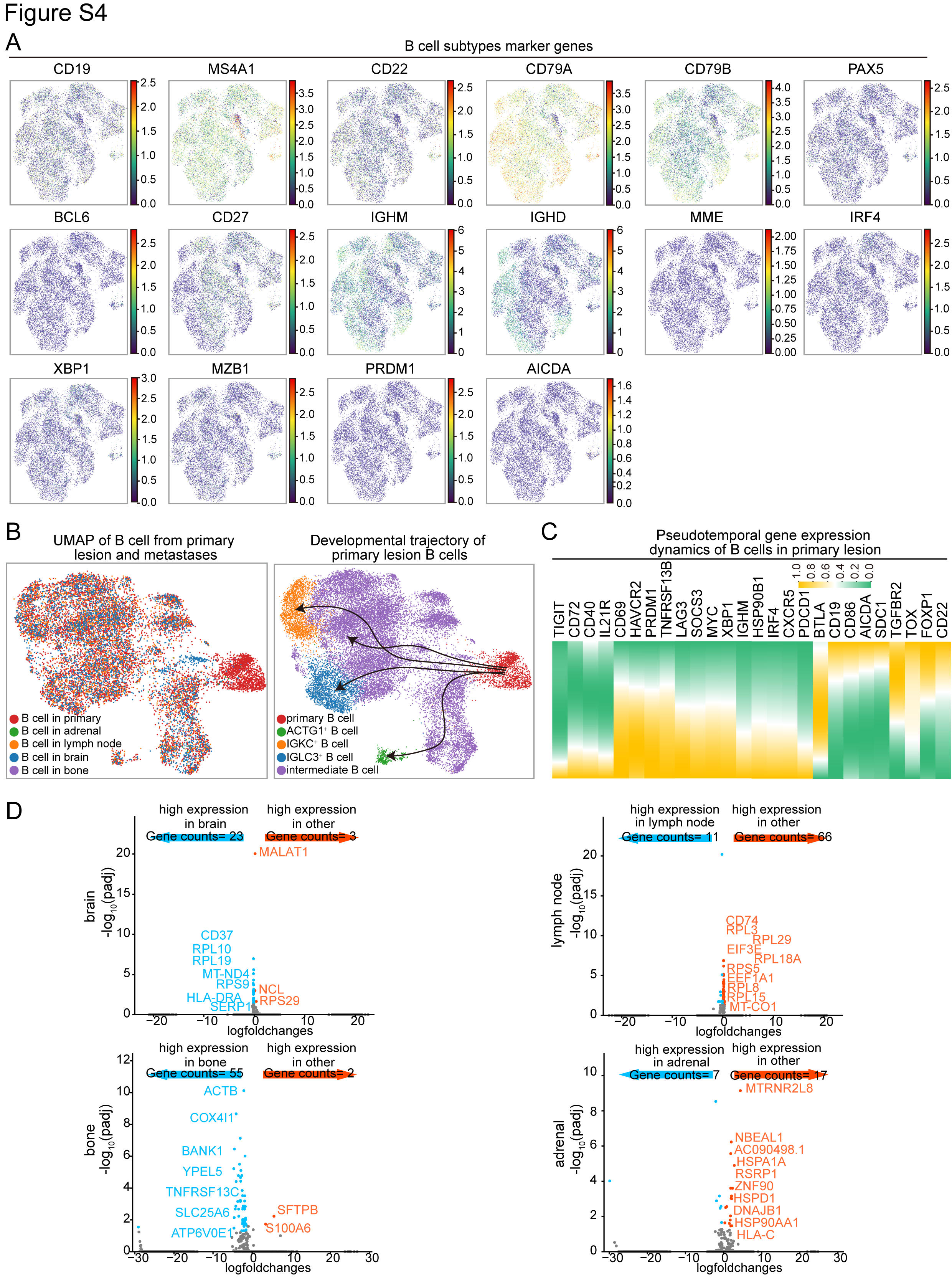

Supplement: Supplementary file 4 — Additional file4UMAP visualization and differential gene expression analysis of B cell subtypes marker genes in metastatic sites. A UMAP visualization depicting the characteristic expression profiles of B cell subtypes marker genes. B UMAP visualization of B cells from four metastatic sitesand pseudotime developmental trajectory using primary lesion B cells as the starting point, with intermediate B cell representing transitional B cell subtype. C Gene expression dynamics of B cells from the primary lesion along pseudotime, with color intensity representing gene expression levels. D A comparative analysis of genes exhibiting elevated expression specifically in B cells from metastatic sites including the brain, lymph node, bone, and adrenal glands, in contrast to the other three metastatic locations. Red indicates genes with high expression in the specific metastasis, while blue represents genes with high expression in the other three metastases [file 12672_2025_2269_MOESM4_ESM.tif]

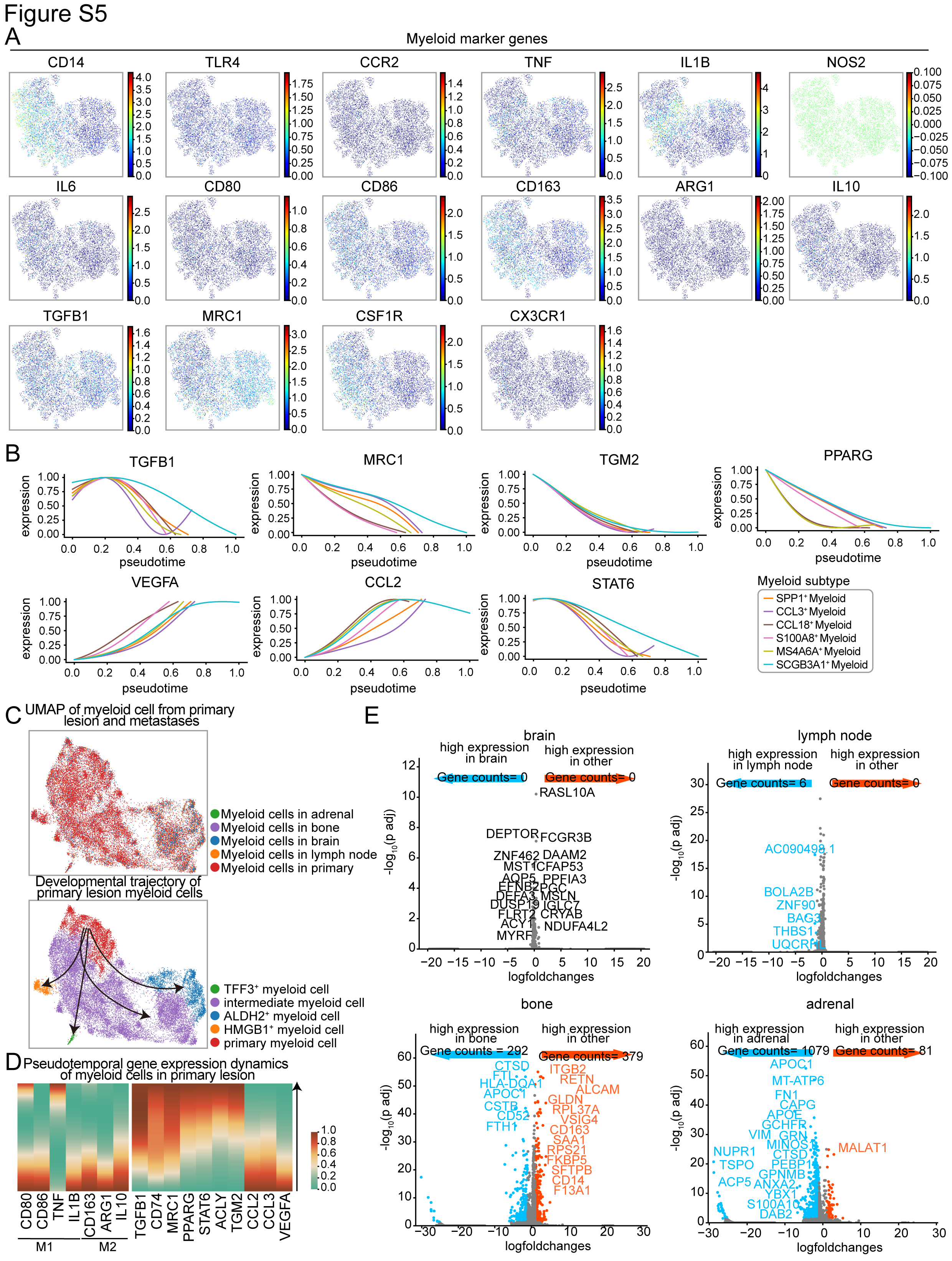

Supplement: Supplementary file 5 — Additional file5UMAP visualization, pseudotime analysis, and differential gene expression in myeloid cells across metastatic sites. A UMAP visualization of marker genes in myeloid cell subpopulations, with red indicating higher expression levels. B Pseudotime trajectory analysis showing the expression patterns of TGFB1, MRC1, TGM2, PPARG, VEGFR, CCL2, and STAT6 in myeloid cell subpopulations. C UMAP visualization of myeloid cells from four metastatic sitesand pseudotime developmental trajectory using primary lesion myeloid cells as the starting point, with intermediate myeloid cell representing transitional myeloid cell subtype. C Gene expression dynamics of myeloid cells from the primary lesion along pseudotime, with color intensity representing gene expression levels. D A comparative analysis of genes exhibiting elevated expression specifically in myeloid cells from metastatic sites including the brain, lymph node, bone, and adrenal glands, in contrast to the other three metastatic locations. Red indicates genes with high expression in the specific metastasis, while blue represents genes with high expression in the other three metastases [file 12672_2025_2269_MOESM5_ESM.tif]

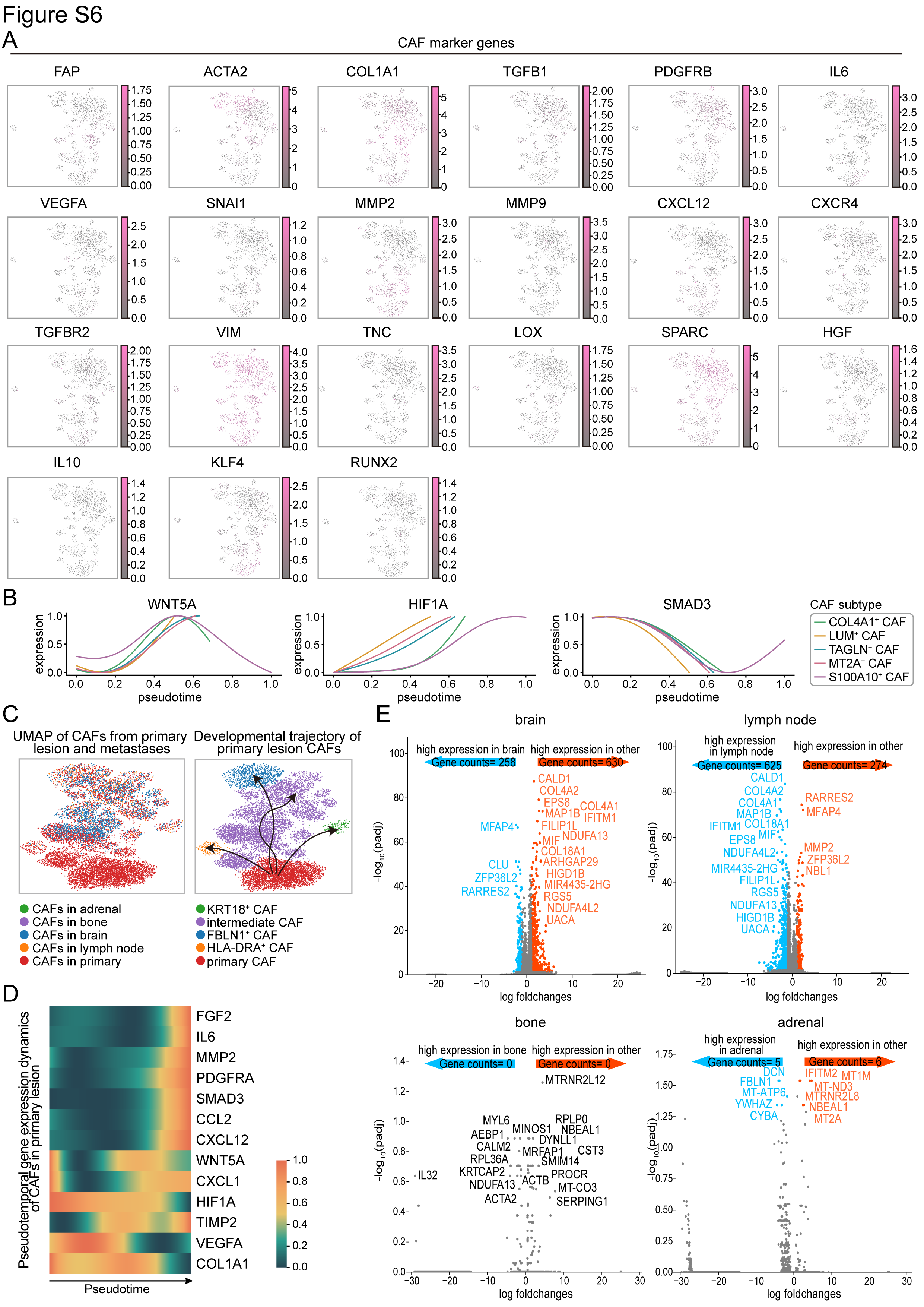

Supplement: Supplementary file 6 — Additional file6UMAP visualization, pseudotime analysis, and differential gene expression in CAFs across metastatic sites. A UMAP visualization of marker genes in CAF subpopulations, with pink indicating higher expression levels. B Pseudotime trajectory analysis showing the expression patterns of WNT5 A, HIF1 A, and SMAD3 in CAF subpopulations. C UMAP visualization of CAFs from four metastatic sitesand pseudotime developmental trajectory using primary lesion CAFs as the starting point, with intermediate CAFs representing transitional CAF subtype. C Gene expression dynamics of CAFs from the primary lesion along pseudotime, with color intensity representing gene expression levels. D A comparative analysis of genes exhibiting elevated expression specifically in CAFs from metastatic sites including the brain, lymph node, bone, and adrenal glands, in contrast to the other three metastatic locations. Pink indicates genes with high expression in the specific metastasis, while grey represents genes with high expression in the other three metastases [file 12672_2025_2269_MOESM6_ESM.tif]

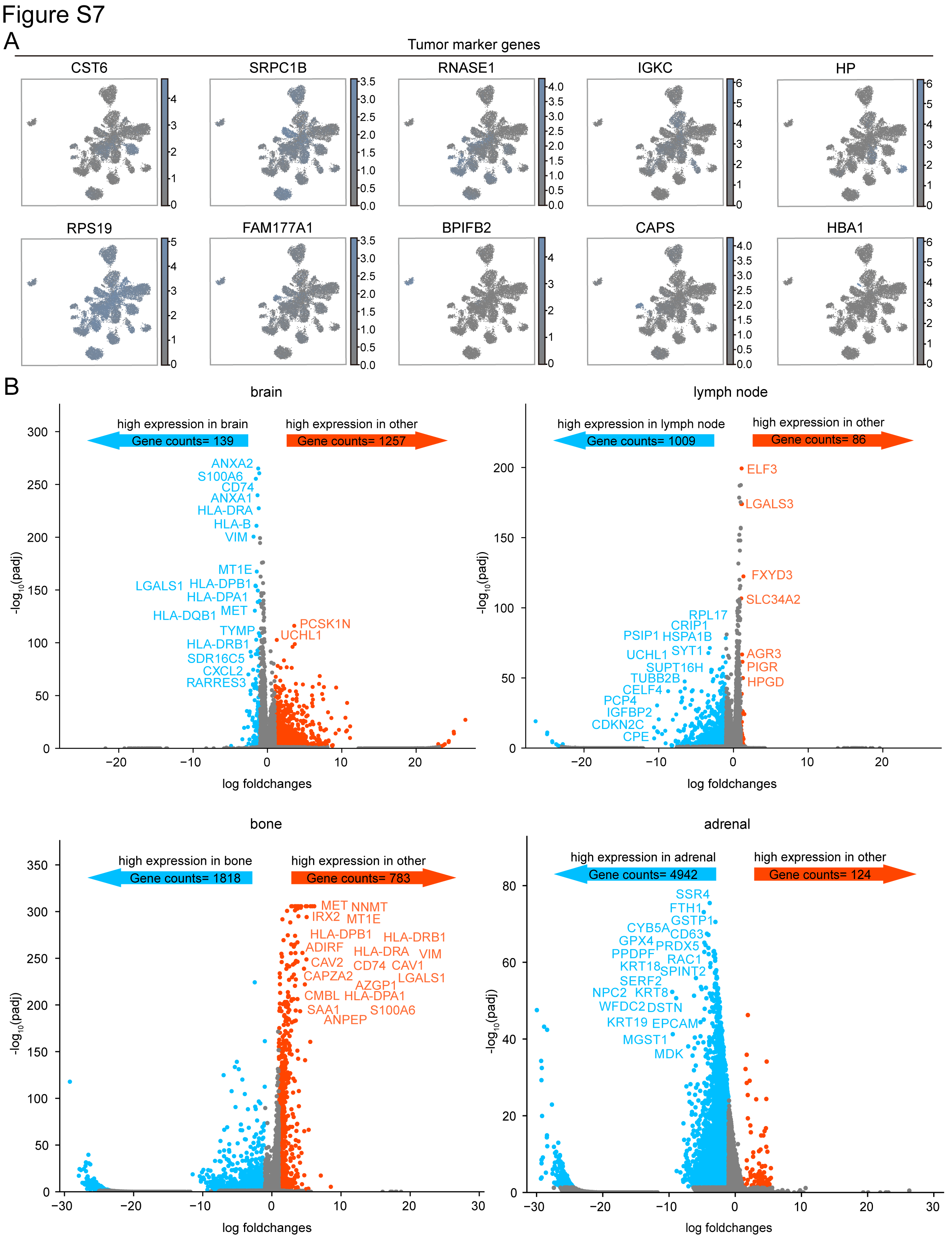

Supplement: Supplementary file 7 — Additional file7UMAP visualization and differential gene expression in tumor cells across metastatic sites. A UMAP visualization of marker genes in tumor cell subpopulations, with blue indicating higher expression levels. B A comparative analysis of genes exhibiting elevated expression specifically in tumor cells from metastatic sites including the brain, lymph node, bone, and adrenal glands, in contrast to the other three metastatic locations. Blue indicates genes with high expression in the specific metastasis, while grey represents genes with high expression in the other three metastases [file 12672_2025_2269_MOESM7_ESM.tif]

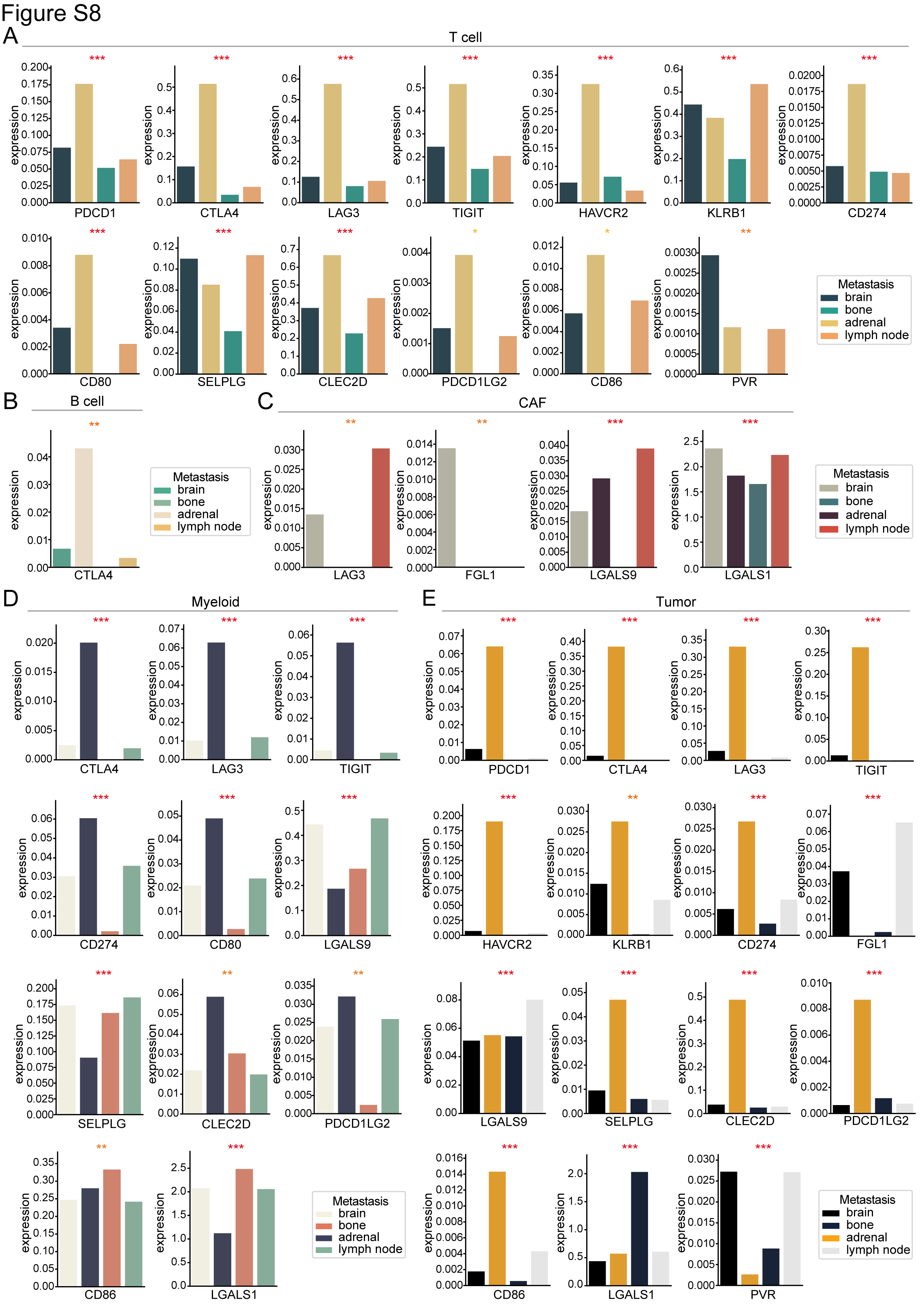

Supplement: Supplementary file 8 — Additional file8Expression of immune checkpoints across immune and tumor cells in all metastatic sites of lung cancer. A Expression of immune checkpoints on T cells across metastatic sites. B Expression of immune checkpoints on B cells. C Expression of immune checkpoints on CAFs. D Expression of immune checkpoints on myeloid cells. E Expression of immune checkpoints on tumor cells. *** Means P < 0.001; ** Means P < 0.01; * Means P < 0.05 [file 12672_2025_2269_MOESM8_ESM.tif]
